# Genome-Wide Characterization and Analysis of bHLH Transcription Factors Related to Crocin Biosynthesis in *Gardenia jasminoides* Ellis (Rubiaceae)

**DOI:** 10.1155/2020/2903861

**Published:** 2020-04-06

**Authors:** Ya Tian, Xiangdong Pu, Haoying Yu, Aijia Ji, Ranran Gao, Yating Hu, Zhichao Xu, Hualei Wang

**Affiliations:** ^1^Key Laboratory of Propagation and Cultivation on Medicinal Plants, College of Agriculture, Guizhou University, Guizhou 550025, China; ^2^Institute of Medicinal Plant Development, Chinese Academy of Medical Science, Peking Union Medical College, Beijing 100193, China; ^3^School of Pharmaceutical Sciences, Guangzhou University of Chinese Medicine, Guangzhou 510006, China; ^4^Engineering Research Center of Chinese Medicine Resource, Ministry of Education, Beijing 100193, China

## Abstract

Crocins, enriched in *Gardenia jasminoides* fruits, have a pharmacological activity against central nervous system diseases, cardiovascular diseases, and cancer cell growth. The biosynthesis of crocins has been widely explored, but its regulatory mechanism remains unknown. Here, the basic helix-loop-helix (bHLH) transcription factors related to crocin biosynthesis were systematically identified on the basis of the genome of *G. jasminoides*. A total of 95 *GjbHLH* transcription factor genes were identified, and their phylogenetic analysis indicated that they could be classified into 23 subfamilies. The combination of gene-specific *bHLH* expression patterns, the coexpression analysis of biosynthesis genes, and the analysis of promoter sequences in crocin biosynthesis pathways suggested that nine *bHLHs* in *G. jasminoides* might negatively regulate crocin biosynthesis. This study laid a foundation for understanding the regulatory mechanism of crocin biosynthesis and the improvement and breeding of *G. jasminoides* varieties.

## 1. Introduction

Crocins, glucosyl esters of crocetin, belong to apocarotenoids, which are highly accumulated in mature fruits of *Gardenia jasminoides* [1] and stigma of *Crocus sativus*. Crocins have a curative effect on various types of central nervous system diseases, such as neurodegenerative diseases [2], and various types of cardiovascular system disease, such as hypertension [3]. Crocins also have pharmacological activities, including anticancer cell growth [4], anti-inflammation and antioxidation [5], antiplatelet aggregation [6], and antiobesity [7]. *G. jasminoides*, named Zhi Zi in traditional Chinese medicine and recorded in the Chinese pharmacopoeia, is commonly used for antioxidant activity, anti-inflammation, and detoxification [8]. *G. jasminoides*, belonging to the coffee family, is a famous ornamental plant, widely distributed around the world [9].

In *G. jasminoides* fruits, the biosynthesis of crocins begins with the cleavage of carotenoids under the activity of carotenoid cleavage dioxygenase (CCD) to produce crocetin dialdehyde. Then, aldehyde dehydrogenase (ALDH) catalyzes the transformation of crocetin dialdehyde to crocetin, which is the key precursor of crocins. Lastly, the glycosylation of different crocetins under the catalysis of UDP-glucosyltransferase (UGT) produces various crocins (crocins I–V) ([Supplementary-material supplementary-material-1]). The genome of *G. jasminoides* has been finished, and the complete pathway of crocin biosynthesis including GjCCD4a, GjALDH2C3, GjUGT94E13, and GjUGT74F8 has been systematically elucidated (NCBI: VZDL00000000). However, the regulation of crocins produced in *G. jasminoides* remains unclear. The regulation of transcription factors (TFs) plays an important role in the biosynthesis of active compounds [10–14].

The bHLH superfamily, one of the largest TF families in plants, has been indicated to be related to plant development and stress response. The genome-wide identification and analysis of bHLH TFs have been reported in many plants, such as *Arabidopsis thaliana* [15], *Oryza sativa* [16], *Solanum lycopersicum* [17], *Brassica rapa* [18], *Salvia miltiorrhiza* [19], *Panax ginseng* [20], *Vitis vinifera* [21], *Malus domestica* [22], and *Arachis hypogaea* [23]. bHLH TFs can bind to E-box (5′-CANNTG-3′), MYCATRD22 (5′-CACATG-3′), T/G-box (5′-AACGTG-3′), or G-box (5′-CACGTG-3′) elements to regulate gene expression [24–26]. Based on transcriptome analysis of *C. sativus*, many transcription factors including two bHLH members were selected to be involved in the crocin biosynthesis and accumulation [27]. Furthermore, the functions of many bHLH proteins in plants have been indicated to regulate the biosynthesis of secondary metabolites. For example, in *Panax notoginseng*, PnbHLH1 improves triterpenoid biosynthesis by interacting with E-box core sequences in the promoter region of target genes [28]. In strawberry fruits, FvbHLH9 positively regulates anthocyanin biosynthesis by forming HY5-bHLH9 transcription complexes [29]. MYCs are important bHLH family members, and MYC2s from different species positively or negatively regulate the biosynthesis of active compounds. In *A. thaliana*, MYC2 negatively regulates indole glucosinolate biosynthesis during jasmonic acid (JA) signaling. Furthermore, MYC2 positively regulates ascorbate redox cycling and flavonoid biosynthesis to enhance tolerance to insect pests and oxidative stress [25]. In *Taxus chinensis*, three JA-inducible MYC TFs, TcJAMYC1, TcJAMYC2, and TcJAMYC4, negatively regulate the paclitaxel biosynthesis [26].

The regulation of bHLH TFs in crocin biosynthesis has not been described in *G. jasminoides*. Here, we systematically selected bHLH TFs and identified the candidate bHLHs related to the regulation of crocin production based on the genome of *G. jasminoides*. Our results provided a basis for further studying the regulatory mechanism of crocin biosynthesis and the breeding of *G. jasminoides* varieties.

## 2. Materials and Methods

### 2.1. Plant Materials

The variety of *G. jasminoides*, named ZZ1-9, was selected and cultivated in the Chongqing Institute of Medicinal Implantation. The root, stem, leaf, young fruit, green fruit, and red fruit of *G. jasminoides* (ZZ1-9) were treated with liquid nitrogen and stored at -80°C for subsequent experiments.

### 2.2. Identification of bHLH Genes and Sequence Feature Analysis

The bHLH sequences of *Arabidopsis* were downloaded from the *Arabidopsis* database (https://www.arabidopsis.org/), Ensembl Plants (http://plants.ensembl.org/index.html), and NCBI (https://www.ncbi.nlm.nih.gov/), and the accession numbers of *AtbHLH* genes are listed in [Supplementary-material supplementary-material-1]. All *GjbHLH* genes were identified using the HMMER analysis of the bHLH domain (HLH: PF00010.26 or bHLH-MYC_N: PF14215.6) against the *G. jasminoides* genome. The *GjbHLH* genes were manually corrected using a protein BLAST algorithm (http://blast.ncbi.nlm.nih.gov/Blast.cgi). The theoretical isoelectric point (p*I*) and the molecular weight (Mw) of the GjbHLH proteins were predicted using the computed p*I*/Mw tool on the ExPASy server (http://web.expasy.org/compute_pi/).

### 2.3. Phylogenetic, Gene Structure, and MEME Motif Analyses

A total of 638 *bHLH* genes from *Arabidopsis*, poplar, rice, moss, and algae were classified into 32 subfamilies [30]. The protein sequences of the bHLH family from *G. jasminoides* and *Arabidopsis* were aligned using MUSCLE methods [31]. Then, the multiple sequence alignments were used to construct a Maximum Likelihood (ML) tree by MEGA 6.0 with the Jones-Taylor-Thornton model and 1000 bootstrap replicates [32]. The phylogenetic tree of the MYC subfamily and the bHLH3 subfamily was constructed using the same method, and bHLH15 subfamily members, *GjbHLH15.1* and *GjbHLH15.7*, were chosen as the outgroup. The online Gene Structure Display Server (GSDS 2.0) (http://gsds.cbi.pku.edu.cn) was used to investigate the gene structure based on each coding sequence (CDS) and the corresponding genomic sequence. The conserved motifs in *bHLH* TFs of *G. jasminoides* were identified using MEME (suite version 5.0.3) with the following criteria: 22 motifs, any number of repetitions of a motif, and an optimum width of 10–200 amino acids [19, 33].

### 2.4. Gene Expression Analysis

The RNA-Seq from six organs (root, stem, leaf, young fruit, green fruit, and red fruit) of *G. jasminoides* was performed. The RNA-Seq reads were mapped to the *G. jasminoides* genome using HISAT2 [34], and the expression levels of *bHLH* genes were estimated on the basis of fragments per kilobase per million (FPKM) values using Cufflinks [35].

Total RNA was extracted from the root, stem, leaves, green fruits, and red fruits using an RNAprep Pure Plant Kit (TIANGEN Biotech, China) according to the manufacturer's instructions. The expression levels of *GjbHLH* candidate genes identified as putative regulators of crocin biosynthesis were confirmed by quantitative real-time reverse transcription PCR (qPCR) analysis in triplicate. Total RNA was reverse transcribed using a PrimeScript™ II 1st Strand cDNA Synthesis Kit (TaKaRa, China). qPCR was performed with a TB Green™ premix Ex Taq™ (Tli RNaseH Plus) (TaKaRa, China) and conducted in triplicate using an Applied Biosystems 7500 Real-Time PCR system (Life Technologies, USA). The primers were designed using Primer Premier 6 ([Supplementary-material supplementary-material-1]), with an amplicon size ranging from 150 bp to 250 bp and an optimal Tm of 55 ± 5°C. The actin gene was used as an internal reference, and red fruit was examined for comparison; Ct values were calculated to analyze the relative expression levels by using the 2^−*ΔΔ*Ct^ method [36]. One-way ANOVA was performed with GraphPad Prism to detect the differences in candidate gene expression. ^∗^*P* < 0.05 was considered to indicate statistical difference in expression.

### 2.5. Promoter Sequence Analysis

The promoter sequences (1500 bp) of key enzyme genes in the crocin biosynthesis pathways were used to predict *cis*-elements in the New PLACE database (https://www.dna.affrc.go.jp/PLACE/).

## 3. Results

### 3.1. Gene Prediction and Phylogenetic Analysis

A total of 95 *bHLHs* were identified that have a complete bHLH domain and named in accordance with the classification principle of *Arabidopsis thaliana* ([Supplementary-material supplementary-material-1]). The GenBank accession numbers of GjbHLH sequences are MN385845 to MN385939 ([Supplementary-material supplementary-material-1]). The length of GjbHLH amino acid (aa) sequences varied from 89 aa (GjbHLH25.9) to 966 aa (GjbHLH23.4). The molecular weights (Mw) of the predicted proteins ranged from 9,904.32 Da (GjbHLH25.9) to 104,750.24 Da (GjbHLH23.4) ([Supplementary-material supplementary-material-1]), and the predicted theoretical isoelectric points (p*I*) ranged from 4.81 (GjbHLH1.5) to 10.51 (GjbHLH31.4) ([Supplementary-material supplementary-material-1]).

**Figure 1:**
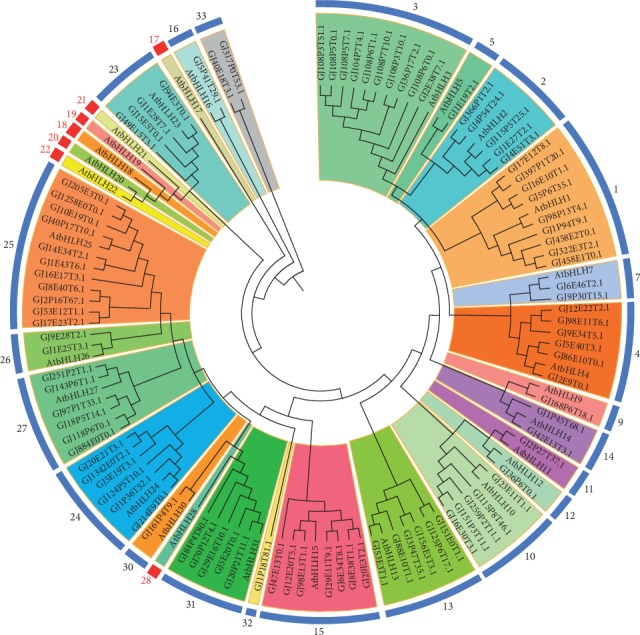
An ML phylogenetic tree of the *bHLH* transcription factor family in *G. jasminoides*. Subfamilies 17, 18, 19, 20, 21, 22, and 28 marked in red indicated those commonly existing in *A. thaliana*, but no *GjbHLH* genes were found in these subfamilies.

The ML phylogenetic tree of the 95 *bHLH* members in *G. jasminoides* indicated that 23 distinct subfamilies were identified (Figure 1). The subfamily 25 had the largest number of members (11 *GjbHLH*s), and subfamilies 5, 9, 11, 12, 16, 30, and 32 had one *GjbHLH* gene each. Subfamilies 17, 18, 19, 20, 21, 22, and 28 commonly exist in *A. thaliana*, but no *GjbHLH* genes were found in these subfamilies. The new subfamilies 32 and 33 were species-specific in *G. jasminoides*.

In addition, 9 genes, which are distributed in subfamilies 2 and 23, possessed a bHLH-MYC_N (Pfam: PF14215.6) domain, and the other genes have a conserved HLH (Pfam: PF00010.26) domain ([Supplementary-material supplementary-material-1]).

### 3.2. Gene Structure and Conserved Motif Analyses

**Figure 2:**
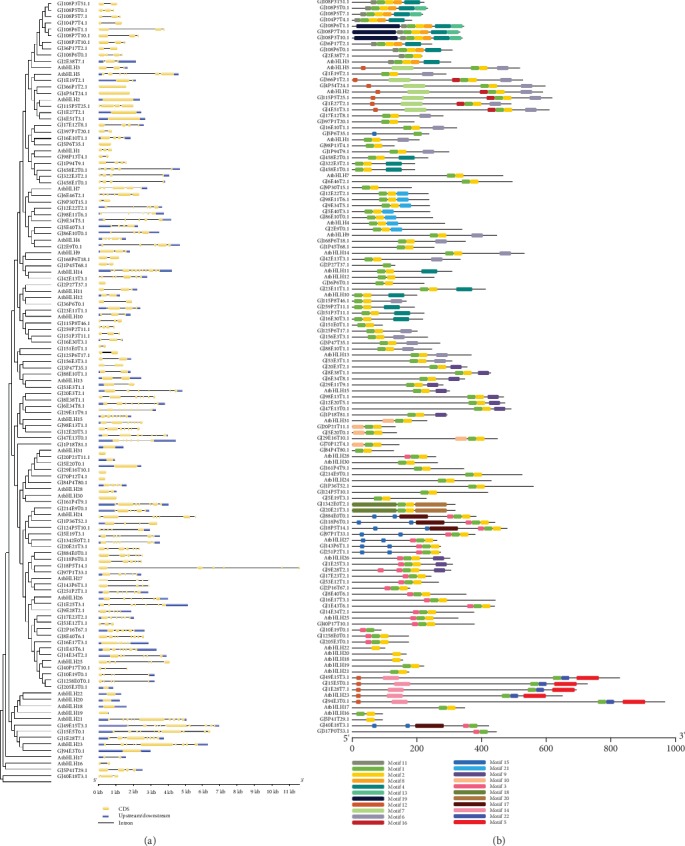
The structural features and conserved motif analysis of *GjbHLH* genes. (a) The structural features of each *bHLH* gene in *G. jasminoides*. Exons and UTRs are represented by yellow and blue round-cornered rectangles, respectively. Introns are shown by black connecting lines. (b) The distribution of conserved motifs in each *bHLH* gene of *G. jasminoides*. The relative positions of each conserved motif within the bHLH protein are shown in color.

The structural analyses of the *bHLH* genes revealed that the number of exons varied from 1 to 12, and 8 genes were intronless (Figure 2(a)). Subfamily 23 had the most number of exons with an average of 12. The 8 intronless genes were distributed across 5 subfamilies. Of these genes, 3 belonged to subfamily 31, 2 belonged to subfamily 2, and 3 belonged to subfamilies 1, 11, and 30 ([Supplementary-material supplementary-material-1]).

**Figure 3:**
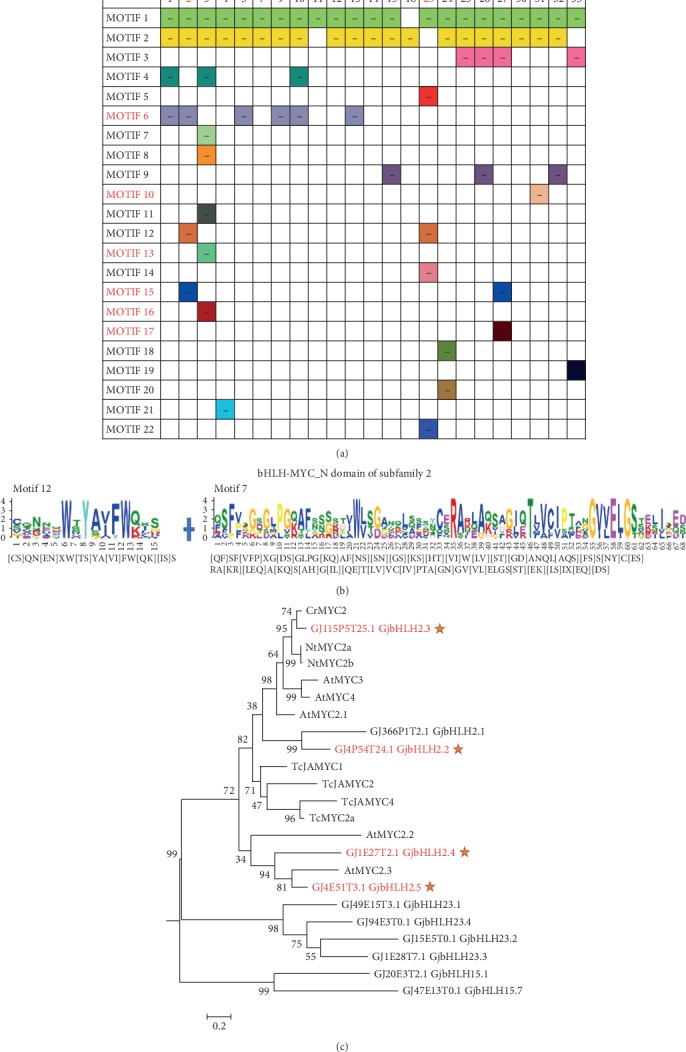
The distribution analysis of conserved motifs of *GjbHLH* genes in each subfamily and the phylogenetic tree and motif analysis of subfamily 2. (a) Distribution of 22 motifs in each subfamily. (b) The bHLH-MYC_N domain of subfamily 2 consists of motifs 12 and 7. (c) An ML phylogenetic tree was constructed with the 12 *MYC2* genes from *C. roseus*, *N. tabacum*, *T. chinensis*, and *A. thaliana* and 9 *MYCs* from *G. jasminoides*. *GjbHLH15.1* and *GjbHLH15.7* branches were chosen as an outgroup. The *MYC2s* were downloaded with the sequence numbers: *CrMYC2* (AF283507); *NtMYC2a* (HM466974) and *NtMYC2b* (HM466975); *TcJAMYC2* (JX519289), *TcJAMYC4* (JX519290), and *TcJAMYC1* (FJ608574); *TcMYC2a* (MG494378); and *AtMYC3* (AT5G46760), *AtMYC4* (AT4G17880), *AtMYC2.1* (AT1G32640), *AtMYC2.2* (AT1G63650), and *AtMYC2.3* (AT1G01260).

The MEME was used to characterize 22 conserved motifs within bHLH proteins in order to clarify the evolution of *bHLH* genes (Figure 2(b) and [Supplementary-material supplementary-material-1]). Compared with the known HLH and bHLH-MYC_N domains, motif 1 and motif 2 belong to the HLH domain, and motifs 7, 12, and 14 belong to the bHLH-MYC_N domain. *GjbHLHs* in subfamilies 2 and 3 contained the largest number of motifs (6 types), whereas the *GjbHLHs* in subfamilies 11 and 16 comprised one motif each (motifs 1 and 2). Additionally, the average number of motifs per *bHLH* varied across subfamilies and ranged from 1 (subfamilies 11 and 16) to 6 (subfamily 2). Most motifs appeared only once in one *bHLH* gene, but GjbHLH26.2 protein had two copies of motif 3, and GjbHLH33.1 protein and subfamily 27 have two copies of motif 15. Motif 1 or 2 was conservatively distributed in all subfamilies. A total of 14 motifs uniquely appear in a specific subfamily: motifs 7 and 16 in subfamily 2; motifs 8, 11, and 13 in subfamily 3; motif 21 in subfamily 4; motifs 5, 14, and 22 in subfamily 23; motifs 18 and 19 in subfamily 24; motif 17 in subfamily 27; motif 10 in subfamily 31; and motif 19 in subfamily 33 (Figure 3(a)).

The bHLH-MYC_N domain of subfamily 2 was composed of motifs 12 and 7, and subfamily 23 comprised motifs 12 and 14 (Figures 3(a) and 3(b)). In addition, the ML phylogenetic tree of MYCs from *Catharanthus roseus*, *Nicotiana tabacum*, *T. chinensis*, and *A. thaliana* and all members of subfamilies 2 and 23 in *G. jasminoides* indicated that all members of subfamily 2 are clustered with MYC2 (Figure 3(c)). Furthermore, *GjbHLH2.3* is clustered with *CrMYC2*, and *GjbHLH2.4* and *GjbHLH2.5* are clustered with *AtMYC2.3*.

### 3.3. Differential Expression of bHLH Genes in Various Organs

**Figure 4:**
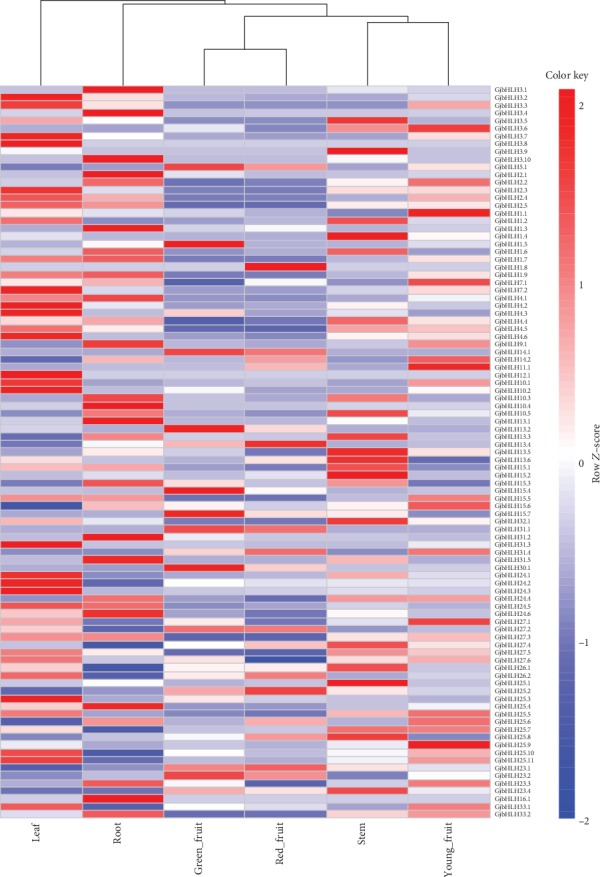
Heatmaps representing the expression profiles of *G. jasminoides bHLH* genes in the root, stem, leaf, young fruit, green fruit, and red fruit. The FPKM value was normalized using *Z*-score (row).

Among the 95 *bHLH* genes, the expression of 13 *bHLH* genes distributed across 8 subfamilies was undetected, with FPKM values of less than 1 (Figure 4, [Supplementary-material supplementary-material-1]). The expression of the other 72 *bHLH* genes comprising 19 subfamilies was detected, with FPKM values higher than 1 in at least one of the six organs tested. In addition, 44 *GjbHLH* genes showed a significantly high expression (log2 |fold change| > 1) in at least one of the organs (root, stem, leaf, and young fruit), and 13 genes exhibited a significantly high expression in all four organs. The expression pattern of *GjbHLH* genes indicated that more *GjbHLH* genes showed a relatively low expression in mature *G. jasminoides* fruits. A total of 13 genes were specifically expressed in six organs. In particular, 7 genes (*GjbHLH1.3*, *GjbHLH2.1*, *GjbHLH3.1*, *GjbHLH3.4*, *GjbHLH3.10*, *GjbHLH10.4*, and *GjbHLH31.2*) were specifically expressed in the root, 2 genes (*GjbHLH15.2* and *GjbHLH25.1*) were specifically expressed in the stem, and only 1 gene was expressed specifically in the leaf (*GjbHLH7.2*), young fruit (*GjbHLH25.9*), green fruit (*GjbHLH15.4*), and red fruit (*GjJbHLH1.8*).

**Figure 5:**
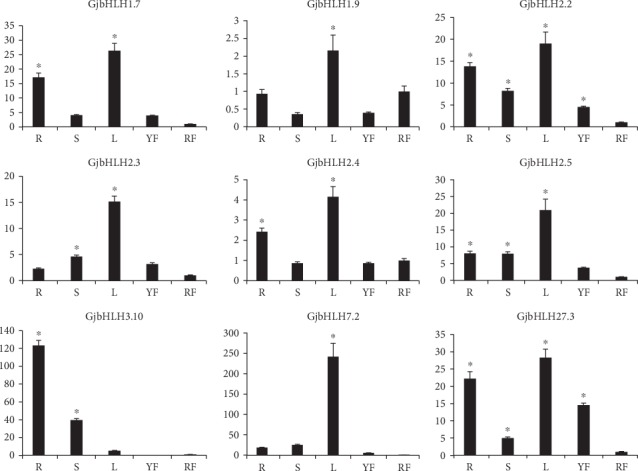
qRT-PCR of nine candidate *bHLH* genes related to the regulation of crocin biosynthesis. The characters on the *x*-axis indicate the root (R), stem (S), leaf (L), young fruit (YF), and red fruit (RF). The *y*-axis represents the relative level of gene expression. The actin gene was used as an internal reference. One-way ANOVA was performed with GraphPad Prism. Asterisks (∗) represent significant differences with the red fruit. ^∗^*P* < 0.05 was considered a significant level.

Crocins have been indicated to be highly accumulated in mature *G. jasminoides* fruits [37]. The expression of the key enzyme genes, including *GjCCD4a*, *GjALDH2C3*, *GjUGT94E13*, and *GjUGT74F8*, in the crocin biosynthesis pathway was higher in mature fruits than in other organs. However, the expression of 9 genes (*GjbHLH1.7*, *GjbHLH1.9*, *GjbHLH2.2*, *GjbHLH2.3*, *GjbHLH2.4*, *GjbHLH2.5*, *GjbHLH3.10*, *GjbHLH7.2*, and *GjbHLH27.3*) significantly decreased in mature fruits compared with those in the four other organs; the FPKM values were higher than 50 in the four other organs and at least 2-fold higher than those in red fruit. Among them, 4 genes (*GjbHLH2.2*, *GjbHLH2.3*, *GjbHLH2.4*, and *GjbHLH2.5*) belonged to the MYC2 family. The qRT-PCR analysis of the 9 candidate genes indicated that the expression of 7 genes in red fruits was significantly low, which was consistent with the RNA-Seq results (Figure 5, [Supplementary-material supplementary-material-1]).

### 3.4. *cis*-Acting Elements of Promoter of Crocin Biosynthetic Genes

The analysis of the promoter sequences of 6 key enzyme genes (*GjBCH*, *GjLCYB*, *GjALDH2C3*, *GjUGT94E13*, *GjUGT74F8*, and *CjCCD4a*) in the crocin biosynthesis pathway and candidate *GjbHLH* genes showed that all the promoters contained E-box sites, which are the regulatory elements of *bHLH* TFs (Tables [Supplementary-material supplementary-material-1]). The promoters of 6 key enzyme genes have 7, 3, 4, 5, 9, and 4 E-box binding sites. In addition, 10 classic MYC2-binding *cis*-acting elements, including 4 AACGTG (T/G-box), 1 CACGTG (G-box), and 5 CACATG (MYCATRD22), were found in the promoters of crocin biosynthetic genes. Moreover, 5 MYCATRD22 elements were found in the promoters of *GjLCYB*, *GjBCH*, *GjALDH2C3*, and *GjUGT74F8*, 4 T/G-box elements were detected in the promoters of *GjALDH2C3*, *GjUGT94E13*, and *GjCCD4a*, and 1 G-box element was observed in the promoter of *GjUGT74E8*. Furthermore, 6 candidates of *GjbHLH* genes (*GjbHLH2.3*, *GjbHLH2.4*, *GjbHLH2.5*, *GjbHLH3.10*, *GjbHLH7.2*, and *GjbHLH27.3*) contained at least one of the elements of T/G-box and MYCATRD22.

## 4. Discussion

bHLH TFs play an important role in plant stress resistance [38], signal transduction [39], secondary metabolism regulation [40], and growth and development [41]. They have been identified and analyzed in various plants. According to the sequence homology and phylogenetic relationships, 167, 183, 177, 169, and 127 bHLH genes have been identified in *A. thaliana* [30], *P. trichocarpa* [30], *O. sativa* [30], *P. ginseng* [20], and *S. miltiorrhiza* [19], respectively. Here, we first identified 95 *GjbHLH* TF genes in *G. jasminoides* and classified them into 23 subfamilies. The gene number of bHLH genes is similar with that in the *V. vinifera* genome (94 genes) [21]. The variable numbers of bHLH genes in these species are probably caused by whole genome duplication or tandem repeat during plant evolution. Both *V. vinifera* and *G. jasminoides* did not experience a whole genome duplication event after the ancient gamma triplication event of eudicots [42, 43]; however, the genomes of *A. thaliana*, *P. trichocarpa*, *O. sativa*, *P. ginseng*, and *S. miltiorrhiza* underwent at least one whole genome duplication event after a shared gamma event [44]. Thence, the bHLH genes in these species showed significant expansion, compared with *V. vinifera* and *G. jasminoides*. In addition, compared with the bHLH family of *Arabidopsis*, the members of subfamilies 17, 18, 19, 20, 21, 22, and 28 of bHLH TFs are lost in the *G. jasminoides* genome, and two new subfamilies 32 and 33 were identified in *G. jasminoides*. Gene families in plants might primarily evolve through tandem duplication [45]. In the *G. jasminoides* genome, we identified one tandem gene duplication, covering 7 *bHLH* genes in scaffold 108. All the clustered *bHLH* genes belonged to subfamily 3, suggesting the expansion of subfamily 3 members in *G. jasminoides*. The phylogenetic analysis of bHLH3 between *G. jasminoides* and previous reports of bHLH3 family members from other plants suggested that the expansion of subfamily 3 was species-specific after the speciation of *G. jasminoides* ([Supplementary-material supplementary-material-1]).

bHLH TFs directly or indirectly regulate the biosynthesis of active compounds in medicinal plants. For example, SmbHLH10 overexpression in *S. miltiorrhiza* enhanced the accumulation of tanshinones [46]. In *Medicago truncatula*, two bHLH TFs, TSARL1 and TSARL2, positively increase the expression of the triterpene biosynthetic genes, resulting in triterpene saponin accumulation [47]. In *G. jasminoides*, the expression profile of 9 candidate *bHLH* TFs in matured fruits significantly decreased and negatively correlated with the expression of *GjCCD4a*, *GjALDH2C3*, *UGT74F8*, and *UGT94E13*. Among them, 4 genes (*GjbHLH2.2*, GjbHLH2*.3*, *GjbHLH2.4*, and *GjbHLH2.5*) had bHLH-MYC_N protein domains. Promoter sequence analysis indicated that the 6 key enzyme genes in crocin biosynthesis pathways had a large number of bHLH TF binding sites, suggesting the potential regulation of *bHLHs* in *G. jasminoides*. The promoters of 7 candidate *GjbHLH* genes contained at least one of the motifs of T/G-box, G-box, and MYCATRD22 that could be bound by MYC2. The phylogenetic analysis of MYCs revealed that *GjbHLH2.3* is clustered with *CrMYC2*, which can regulate the biosynthesis of medicinally valuable terpenoid indole alkaloids in *C. roseus* [48]. Furthermore, the qPCR analysis indicated that the expression of most candidate genes in mature fruits was significantly low, which was consistent with the RNA-Seq results. These results implied that *bHLH* TFs might negatively regulate the biosynthesis of crocins in *G. jasminoides*. Candidate *bHLH* TFs should be functionally verified to clarify the molecular mechanism of the regulation of crocin biosynthesis.

## 5. Conclusions

In conclusion, 95 *bHLH* TF genes were identified and phylogenetic analyzed in the genome of *G. jasminoides*. These *GjbHLHs* could be classified into 23 subfamilies supported by phylogeny, additional protein motifs, and intron/exon structures. Gene-specific expression patterns, crocin biosynthesis pathways, and elucidation of the complete pathway of the crocin biosynthesis in mature *G. jasminoides* fruits revealed that 9 *bHLH* TF genes identified in *G. jasminoides* were potentially involved in the regulation of crocin biosynthesis. The candidate *bHLH* TF genes related to crocin biosynthesis should be further functionally identified via a series of *in vivo* and *in vitro* experimental procedures.

## Data Availability

The bHLH transcription factor sequences of *G. jasminoides* have been uploaded into NCBI database, and the GenBank accession numbers of GjbHLH sequences are from MN385845 to MN385939.

## Supplementary Materials

Supplementary 1 Figure S1: the potential crocin biosynthetic pathway in *G. jasminoides*.

Supplementary 2 Figure S2: an ML phylogenetic tree was constructed with *GjbHLH3* subfamily and bHLH3 family members reported from other plants. *GjbHLH15.1* and *GjbHLH15.7* branches were chosen as the outgroup. bHLH3 family members were downloaded with the following sequence numbers: AT2G22770, AT2G22750, AT2G22760, AT4G37850, OS03G51580, OS03G12760, OS03G46860, OS12G43620, OS03G46790, and OS10G01530.

Supplementary 3 Table S1: the accession numbers of *AtbHLH* genes. Table S2: primer sequences of candidate *GjbHLH* genes. Table S3: identification of *GjbHLH* genes through BLAST and HMMER analysis of the *bHLH* domain against the *G. jasminoides* genome. Table S4: analysis of the structural features and conserved motifs of *bHLH* genes in *G. jasminoides*. Table S5: conserved motifs predicted by MEME. Table S6: the FPKM values of 95 *GjbHLH* genes in different organs. Asterisks (∗) represent the coexpression of four genes (*GjALDH2C3*, *GjUGT94E13*, *GjUGT74F8*, and *CjCCD4a*) in crocin biosynthesis. Table S7: the qRT-PCR analysis for candidate *GjbHLH* genes. Table S8: the *cis*-acting elements of the promoter sequences of 6 key enzyme genes (*GjBCH*, *GjLCYB*, *GjALDH2C3*, *GjUGT94E13*, *GjUGT74F8*, and *CjCCD4a*) in the crocin biosynthesis pathway. Table S9: the *cis*-acting elements of the promoter sequences of candidate *GjbHLH* genes.
