# Supplementary material for: Genome-Wide Characterization and Analysis of bHLH Transcription Factors Related to Crocin Biosynthesis in Gardenia jasminoides Ellis (Rubiaceae)
Source: Biomed Res Int. 2020 Apr 6;2020:2903861. doi: 10.1155/2020/2903861 (PMC7165322; doi:10.1155/2020/2903861)
Supplement: Supplementary 1 — Figure S1: the potential crocin biosynthetic pathway in G. jasminoides. [file 2903861.f1.pptx]

## Slide 1
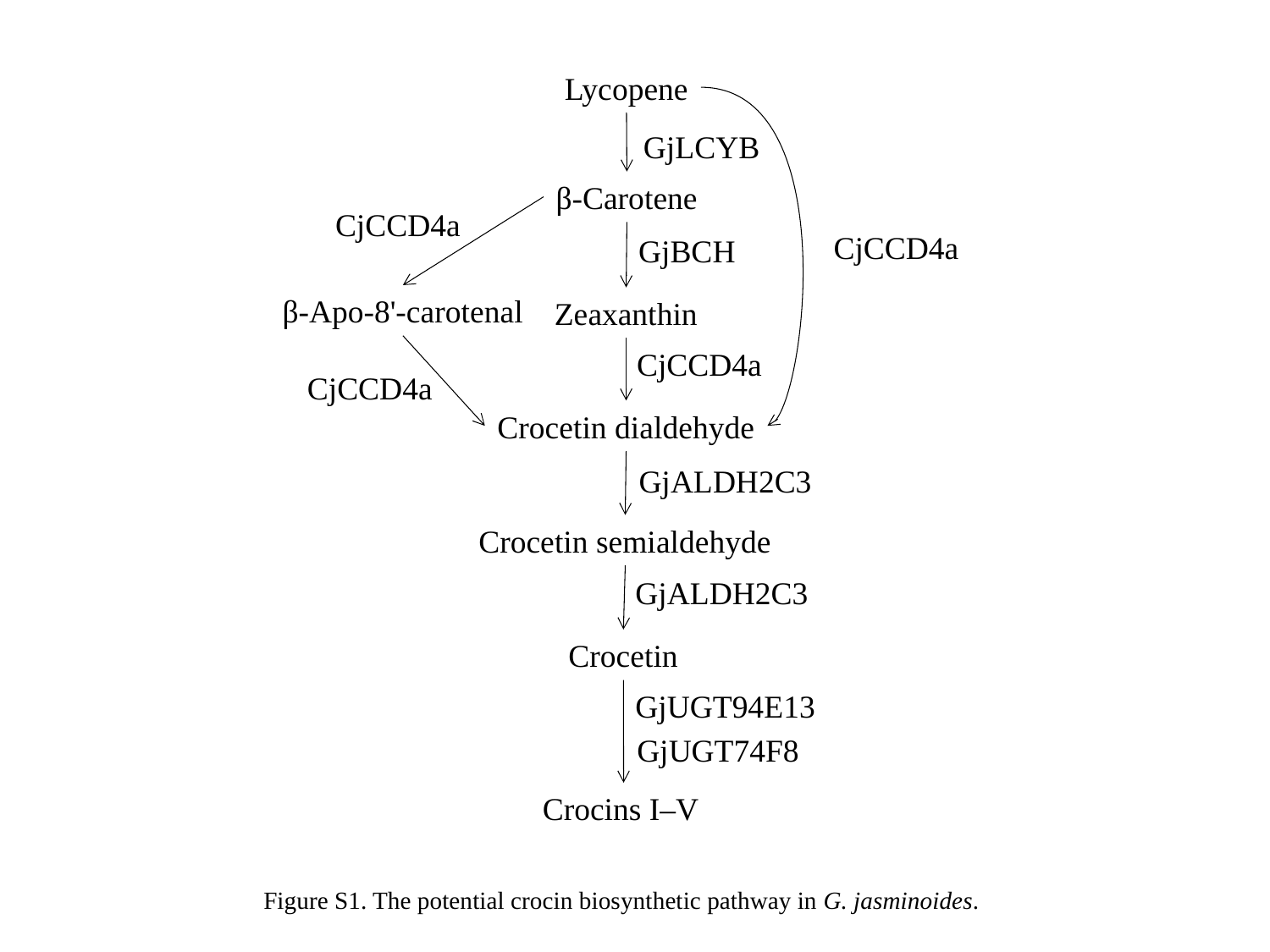

Lycopene
GjLCYB
β-Carotene
CjCCD4a
CjCCD4a
GjBCH
β-Apo-8'-carotenal
Zeaxanthin
CjCCD4a
CjCCD4a
Crocetin dialdehyde
GjALDH2C3
Crocetin semialdehyde
GjALDH2C3
Crocetin
GjUGT94E13
GjUGT74F8
Crocins I–V
Figure S1. The potential crocin biosynthetic pathway in G. jasminoides.
