# Supplementary material for: Genome-Wide Characterization and Analysis of bHLH Transcription Factors Related to Crocin Biosynthesis in Gardenia jasminoides Ellis (Rubiaceae)
Source: Biomed Res Int. 2020 Apr 6;2020:2903861. doi: 10.1155/2020/2903861 (PMC7165322; doi:10.1155/2020/2903861)
Supplement: Supplementary 2 — Figure S2: an ML phylogenetic tree was constructed with GjbHLH3 subfamily and bHLH3 family members reported from other plants. GjbHLH15.1 and GjbHLH15.7 branches were chosen as the outgroup. bHLH3 family members were downloaded with the following sequence numbers: AT2G22770, AT2G22750, AT2G22760, AT4G37850, OS03G51580, OS03G12760, OS03G46860, OS12G43620, OS03G46790, and OS10G01530. [file 2903861.f2.pptx]

## Slide 1
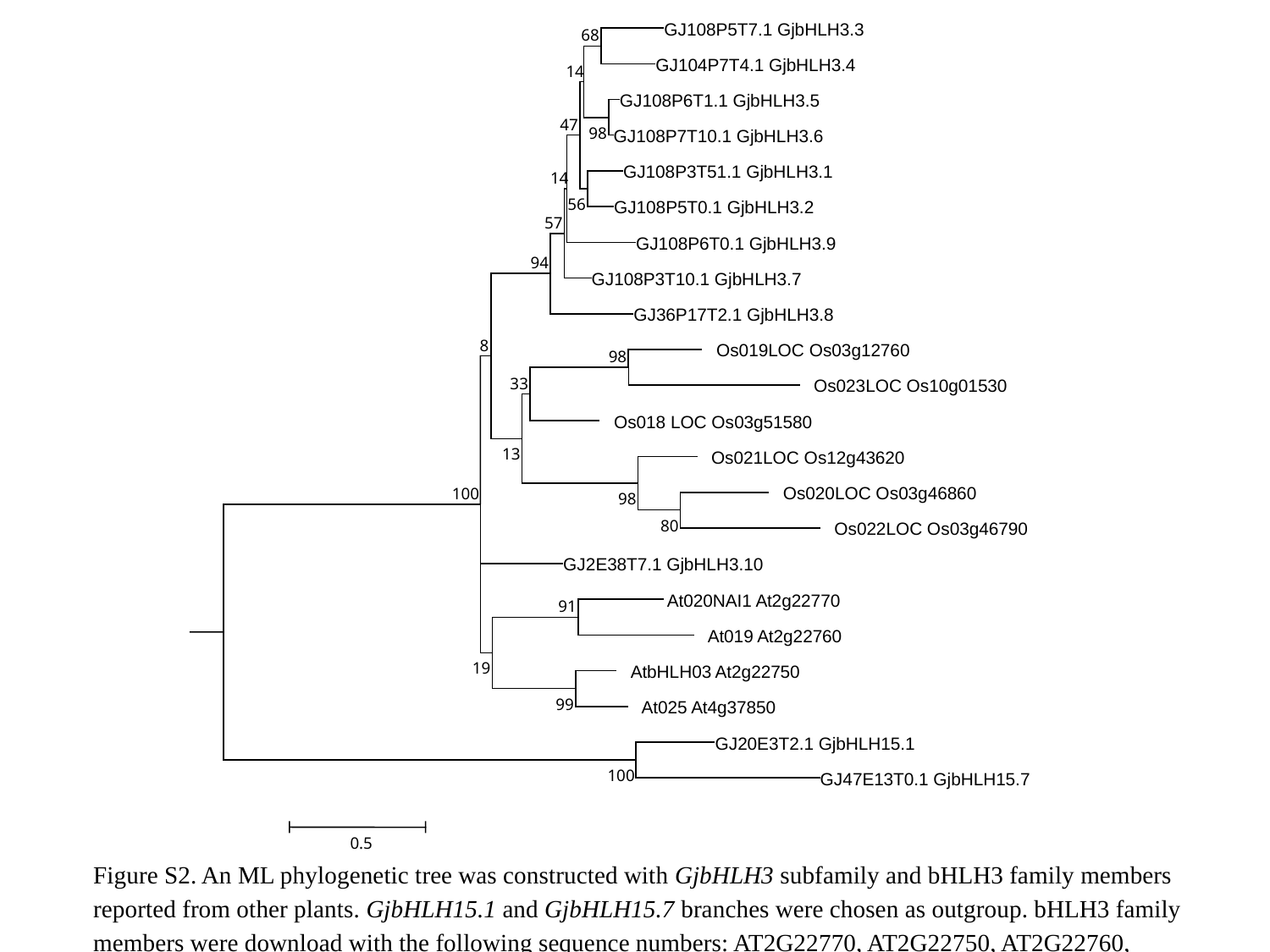

GJ108P5T7.1 GjbHLH3.3
68
 GJ104P7T4.1 GjbHLH3.4
14
 GJ108P6T1.1 GjbHLH3.5
47
98
 GJ108P7T10.1 GjbHLH3.6
 GJ108P3T51.1 GjbHLH3.1
14
56
 GJ108P5T0.1 GjbHLH3.2
57
 GJ108P6T0.1 GjbHLH3.9
94
 GJ108P3T10.1 GjbHLH3.7
 GJ36P17T2.1 GjbHLH3.8
8
 Os019LOC Os03g12760
98
33
 Os023LOC Os10g01530
 Os018 LOC Os03g51580
13
 Os021LOC Os12g43620
 Os020LOC Os03g46860
100
98
80
 Os022LOC Os03g46790
 GJ2E38T7.1 GjbHLH3.10
 At020NAI1 At2g22770
91
 At019 At2g22760
19
 AtbHLH03 At2g22750
99
 At025 At4g37850
 GJ20E3T2.1 GjbHLH15.1
100
 GJ47E13T0.1 GjbHLH15.7
0.5
Figure S2. An ML phylogenetic tree was constructed with GjbHLH3 subfamily and bHLH3 family members reported from other plants. GjbHLH15.1 and GjbHLH15.7 branches were chosen as outgroup. bHLH3 family members were download with the following sequence numbers: AT2G22770, AT2G22750, AT2G22760, AT4G37850, OS03G51580, OS03G12760, OS03G46860, OS12G43620, OS03G46790, and OS10G01530.
