# Supplementary material for: Genome-Wide Characterization and Analysis of bHLH Transcription Factors Related to Crocin Biosynthesis in Gardenia jasminoides Ellis (Rubiaceae)
Source: Biomed Res Int. 2020 Apr 6;2020:2903861. doi: 10.1155/2020/2903861 (PMC7165322; doi:10.1155/2020/2903861)
Supplement: Supplementary 3 — Table S1: the accession numbers of AtbHLH genes. Table S2: primer sequences of candidate GjbHLH genes. Table S3: identification of GjbHLH genes through BLAST and HMMER analysis of the bHLH domain against the G. jasminoides genome. Table S4: analysis of the structural features and conserved motifs of bHLH genes in G. jasminoides. Table S5: conserved motifs predicted by MEME. Table S6: the FPKM values of 95 GjbHLH genes in different organs. Asterisks (∗) represent the coexpression of four genes (GjALDH2C3, GjUGT94E13, GjUGT74F8, and CjCCD4a) in crocin biosynthesis. Table S7: the qRT-PCR analysis for candidate GjbHLH genes. Table S8: the cis-acting elements of the promoter sequences of 6 key enzyme genes (GjBCH, GjLCYB, GjALDH2C3, GjUGT94E13, GjUGT74F8, and CjCCD4a) in the crocin biosynthesis pathway. Table S9: the cis-acting elements of the promoter sequences of candidate GjbHLH genes. [file 2903861.f3.pdf]

Table S1. The accession numbers of *AtbHLH* genes.

sequenc ID	gene ID	sequenc ID	gene ID
AtbHLH1	AT4G21330	AtbHLH17	AT5G64340
AtbHLH2	AT4G17880	AtbHLH18	AT2G43060
AtbHLH3	AT2G22750	AtbHLH19	AT3G06590
AtbHLH4	AT4G36060	AtbHLH20	AT5G57780
AtbHLH5	AT4G09820	AtbHLH21	AT2G18969
AtbHLH7	AT5G56960	AtbHLH22	AT3G29370
AtbHLH9	AT2G31220	AtbHLH23	AT2G27230
AtbHLH10	AT3G06120	AtbHLH24	AT2G43010
AtbHLH11	AT1G49770	AtbHLH25	AT2G42300
AtbHLH12	AT3G56970	AtbHLH26	AT1G03040
AtbHLH13	AT1G68810	AtbHLH27	AT4G09180
AtbHLH14	AT5G08130	AtbHLH28	AT1G27740
AtbHLH15	AT4G21340	AtbHLH30	AT1G30670
AtbHLH16	AT5G15160	AtbHLH31	AT3G50330

Table S2. Primer sequences of candidate GjbHLH genes.

subfamily	sequence ID	GenBank accession number	F	R
1	GjbHLH1.7	MN385867	AATGATCCAGACAGCCCTATGA	CAGCCTCATCACCACAGTAGA
	GjbHLH1.9	MN385869	CTGAGCAAGCCTGTGATT	TCGCCTCTAAGAATACTGTC
2	GjbHLH2.2	MN385857	CCACCACAGCATCATCAT	ATCGCATCCATCAACCTG
	GjbHLH2.3	MN385858	AGGAATTGAGGAACCAGATAG	TGTAGCAACTCCAGGTCTA
	GjbHLH2.4	MN385859	ATCCAAGAAGTTGCTCTGAA	ACGACATTAGGTGCGATAG
	GjbHLH2.5	MN385860	AGTGACGAGGATAGTTATGC	CCGACATCAGTTGGAATCA
3	GjbHLH3.10	MN385854	CAATAGACCGATGAAGCAGCAA	ACAGAAGACATGGTTTCCTCCT
7	GjbHLH7.2	MN385871	AGAAGGGAGAGGCAGAAG	CCAGATGAAGGATGAACCA
27	GjbHLH27.3	MN385916	GGCTTGAACAATCGTCTTAA	GGCTATTGAATGGAGAATCG
	Actin		TCCTCTTCCAGCCTTCTATCAT	GAACCACCACTGAGCACAAT

Table S3. Identification of GjbHLH genes through BLAST and HMMER analysis of the bHLH domain against the G. jasminoides genome.

subfamily	gene ID	sequen ID	GenBank accession number	alignment		envelope		hmm acc/accession	Name	HMM			bit score	Domain E-values		Method
				start	end	start	end			start	end	length		Individual	Conditional	
3	GJ108P3T51.1	GjbHLH3.1	MN385845	73	118	71	118	PF00010.26	HLH	5	53	53	36.71	3.00E-09	1.70E-13	pfam
	GJ108P5T0.1	GjbHLH3.2	MN385846	56	101	53	101	PF00010.26	HLH	5	53	53	40	2.80E-10	1.60E-14	pfam
	GJ108P5T7.1	GjbHLH3.3	MN385847	41	86	39	86	PF00010.26	HLH	5	53	53	38.32	9.30E-10	5.20E-14	pfam
	GJ104P7T4.1	GjbHLH3.4	MN385848	23	68	20	68	PF00010.26	HLH	5	53	53	36.97	2.50E-09	1.40E-13	pfam
	GJ108P6T1.1	GjbHLH3.5	MN385849	166	212	163	212	PF00010.26	HLH	4	53	53	34.66	1.30E-08	7.20E-13	pfam
	GJ108P7T10.1	GjbHLH3.6	MN385850	155	200	152	200	PF00010.26	HLH	5	53	53	34.82	1.20E-08	6.40E-13	pfam
	GJ108P3T10.1	GjbHLH3.7	MN385851	164	209	161	209	PF00010.26	HLH	5	53	53	42.05	6.40E-11	3.60E-15	pfam
	GJ36P17T2.1	GjbHLH3.8	MN385852	74	119	71	119	PF00010.26	HLH	5	53	53	39.69	3.50E-10	1.90E-14	pfam
	GJ108P6T0.1	GjbHLH3.9	MN385853	141	186	138	186	PF00010.26	HLH	5	53	53	27.47	2.30E-06	1.30E-10	pfam
	GJ2E38T7.1	GjbHLH3.10	MN385854	158	205	156	205	PF00010.26	HLH	3	53	53	42.02	6.50E-11	3.60E-15	pfam
5	GJ1E19T2.1	GjbHLH5.1	MN385855	85	132	84	132	PF00010.26	HLH	3	53	53	49	4.30E-13	2.40E-17	pfam
2	GJ366P1T2.1	GjbHLH2.1	MN385856	4	192	1	192	PF14215.6	bHLH-MYC_N	10	175	175	141.47	3.30E-41	3.70E-45	pfam
				330	375	328	376	PF00010.26	HLH	4	52	53	40.67	1.70E-10	1.90E-14	pfam
	GJ4P54T24.1	GjbHLH2.2	MN385857	35	238	35	238	PF14215.6	bHLH-MYC_N	1	175	175	164.52	2.80E-48	3.10E-52	pfam
				393	439	391	439	PF00010.26	HLH	4	53	53	41.44	9.90E-11	1.10E-14	pfam
	GJ115P5T25.1	GjbHLH2.3	MN385858	48	233	48	233	PF14215.6	bHLH-MYC_N	1	175	175	182.75	6.90E-54	7.70E-58	pfam
				437	482	435	483	PF00010.26	HLH	4	52	53	41.56	9.10E-11	1.00E-14	pfam
	GJ1E27T2.1	GjbHLH2.4	MN385859	49	227	48	228	PF14215.6	bHLH-MYC_N	2	173	175	147.17	5.90E-43	6.50E-47	pfam
				344	390	342	390	PF00010.26	HLH	4	53	53	41.57	9.00E-11	1.00E-14	pfam
	GJ4E51T3.1	GjbHLH2.5	MN385860	53	241	53	241	PF14215.6	bHLH-MYC_N	1	175	175	190.88	2.20E-56	2.50E-60	pfam
				440	486	438	486	PF00010.26	HLH	4	53	53	41.23	1.10E-10	1.30E-14	pfam
1	GJ17E12T8.1	GjbHLH1.1	MN385861	92	134	88	135	PF00010.26	HLH	7	52	53	38.55	7.90E-10	4.40E-14	pfam

	GJ397P1T20.1	GjbHLH1.2	MN385862	89	131	85	132	PF00010.26	HLH	7	52	53	40.2	2.40E-10	1.30E-14	pfam
	GJ16E10T1.1	GjbHLH1.3	MN385863	156	199	151	200	PF00010.26	HLH	6	52	53	38.16	1.00E-09	5.80E-14	pfam
	GJ5P6T35.1	GjbHLH1.4	MN385864	188	214	183	220	PF00010.26	HLH	6	38	53	25.36	1.00E-05	5.80E-10	pfam
	GJ98P13T4.1	GjbHLH1.5	MN385865	38	84	36	84	PF00010.26	HLH	4	53	53	43.77	1.80E-11	1.00E-15	pfam
	GJ1P94T9.1	GjbHLH1.6	MN385866	115	158	109	158	PF00010.26	HLH	7	53	53	34.85	1.10E-08	6.30E-13	pfam
	GJ458E2T0.1	GjbHLH1.7	MN385867	48	91	43	91	PF00010.26	HLH	7	53	53	43.92	1.70E-11	9.30E-16	pfam
	GJ322E3T2.1	GjbHLH1.8	MN385868	6	51	3	51	PF00010.26	HLH	5	53	53	37.72	1.40E-09	8.00E-14	pfam
	GJ458E1T0.1	GjbHLH1.9	MN385869	6	51	3	51	PF00010.26	HLH	5	53	53	37.72	1.40E-09	8.00E-14	pfam
7	GJ6E46T2.1	GjbHLH7.1	MN385870	335	381	333	381	PF00010.26	HLH	4	53	53	46.81	2.10E-12	1.20E-16	pfam
	GJ9P30T15.1	GjbHLH7.2	MN385871	25	72	23	72	PF00010.26	HLH	3	53	53	39	5.70E-10	3.20E-14	pfam
4	GJ12E22T2.1	GjbHLH4.1	MN385872	78	125	76	126	PF00010.26	HLH	4	52	53	39.97	2.90E-10	1.60E-14	pfam
	GJ98E11T6.1	GjbHLH4.2	MN385873	80	126	77	127	PF00010.26	HLH	5	52	53	35.77	5.80E-09	3.30E-13	pfam
	GJ9E34T5.1	GjbHLH4.3	MN385874	87	135	84	135	PF00010.26	HLH	4	53	53	35.18	8.90E-09	5.00E-13	pfam
	GJ5E40T3.1	GjbHLH4.4	MN385875					pfam00010	HLH	31	80			7.45E-05		BLASTP
	GJ86E10T0.1	GjbHLH4.5	MN385876					pfam00010	HLH	39	88			3.43E-03		BLASTP
	GJ2E9T0.1	GjbHLH4.6	MN385877	57	104	57	106	PF00010.26	HLH	1	50	53	36.49	3.50E-09	1.90E-13	pfam
9	GJ168P6T18.1	GjbHLH9.1	MN385878	137	183	135	184	PF00010.26	HLH	3	52	53	39.59	3.70E-10	2.10E-14	pfam
14	GJ1P45T68.1	GjbHLH14.1	MN385879	137	180	132	180	PF00010.26	HLH	7	53	53	46.21	3.20E-12	1.80E-16	pfam
	GJ42E13T3.1	GjbHLH14.2	MN385880	32	81	31	81	PF00010.26	HLH	2	53	53	58.29	5.40E-16	3.00E-20	pfam
11	GJ2P27T37.1	GjbHLH11.1	MN385881	94	120	89	129	PF00010.26	HLH	7	33	53	23.11	5.30E-05	2.90E-09	pfam
12	GJ36P6T0.1	GjbHLH12.1	MN385882	65	117	65	117	PF00010.26	HLH	1	53	53	46.09	3.50E-12	1.90E-16	pfam
10	GJ23E11T1.1	GjbHLH10.1	MN385883	203	254	203	254	PF00010.26	HLH	1	53	53	39.25	4.80E-10	2.70E-14	pfam
	GJ115P8T46.1	GjbHLH10.2	MN385884	2	50	1	50	PF00010.26	HLH	4	53	53	40.13	2.50E-10	1.40E-14	pfam
	GJ259P2T11.1	GjbHLH10.3	MN385885	2	50	1	50	PF00010.26	HLH	4	53	53	41.38	1.00E-10	5.80E-15	pfam
	GJ151P3T11.1	GjbHLH10.4	MN385886	22	73	22	73	PF00010.26	HLH	1	53	53	42.79	3.70E-11	2.10E-15	pfam
	GJ16E30T3.1	GjbHLH10.5	MN385887	2	50	1	50	PF00010.26	HLH	4	53	53	34.46	1.50E-08	8.30E-13	pfam

	GJ151E0T1.1	GjbHLH13.1	MN385888	11	58	9	58	PF00010.26	HLH	3	53	53	44.24	1.30E-11	7.40E-16	pfam
	GJ125P6T17.1	GjbHLH13.2	MN385889	40	85	38	86	PF00010.26	HLH	4	52	53	34.1	1.90E-08	1.10E-12	pfam
13	GJ156E3T3.1	GjbHLH13.3	MN385890	44	89	42	90	PF00010.26	HLH	4	52	53	40.89	1.50E-10	8.20E-15	pfam
	GJ3P47T35.1	GjbHLH13.4	MN385891	80	126	78	127	PF00010.26	HLH	3	52	53	47.2	1.60E-12	8.80E-17	pfam
	GJ88E10T1.1	GjbHLH13.5	MN385892	73	119	71	120	PF00010.26	HLH	3	52	53	46.45	2.70E-12	1.50E-16	pfam
	GJ53E3T1.1	GjbHLH13.6	MN385893	173	220	171	220	PF00010.26	HLH	3	53	53	46.81	2.10E-12	1.20E-16	pfam
	GJ20E3T2.1	GjbHLH15.1	MN385894					pfam00010	HLH	241	281			4.92E-03		BLASTP
	GJ8E38T1.1	GjbHLH15.2	MN385895					pfam00010	HLH	320	360			2.22E-03		BLASTP
	GJ6E34T8.1	GjbHLH15.3	MN385896					pfam00010	HLH	210	250			4.46E-03		BLASTP
15	GJ29E11T9.1	GjbHLH15.4	MN385897					pfam00010	HLH	169	209			3.30E-03		BLASTP
	GJ98E13T1.1	GjbHLH15.5	MN385898					pfam00010	HLH	358	398			1.01E-03		BLASTP
	GJ12E20T5.1	GjbHLH15.6	MN385899					pfam00010	HLH	362	402			3.90E-04		BLASTP
	GJ47E13T0.1	GjbHLH15.7	MN385900					cd00083	HLH	383	423			6.56E-03		BLASTP
32	GJ1P18T81.1	GjbHLH32.1	MN385901	165	213	164	213	PF00010.26	HLH	2	53	53	30.71	2.20E-07	1.20E-11	pfam
	GJ20P21T11.1	GjbHLH31.1	MN385902	40	80	32	81	PF00010.26	HLH	9	52	53	30.8	2.10E-07	1.20E-11	pfam
	GJ5E20T0.1	GjbHLH31.2	MN385903	43	83	35	84	PF00010.26	HLH	9	52	53	29.17	6.70E-07	3.70E-11	pfam
	GJ29E16T10.1	GjbHLH31.3	MN385904	360	399	355	400	PF00010.26	HLH	10	52	53	26.73	3.90E-06	2.20E-10	pfam
	GJ70P12T4.1	GjbHLH31.4	MN385905	41	80	36	81	PF00010.26	HLH	10	52	53	30.82	2.10E-07	1.10E-11	pfam
	GJ84P4T80.1	GjbHLH31.5	MN385906	9	50	4	51	PF00010.26	HLH	8	52	53	35.68	6.20E-09	3.50E-13	pfam
30	GJ161P4T9.1	GjbHLH30.1	MN385907					cd00083		196	237			5.20E-04		BLASTP
	GJ214E9T0.1	GjbHLH24.1	MN385908	346	392	344	392	PF00010.26	HLH	4	53	53	54.95	6.00E-15	3.30E-19	pfam
	GJ1P36T52.1	GjbHLH24.2	MN385909	355	401	353	401	PF00010.26	HLH	4	53	53	50.6	1.40E-13	7.60E-18	pfam
24	GJ124P5T10.1	GjbHLH24.3	MN385910	208	253	205	253	PF00010.26	HLH	5	53	53	51.65	6.40E-14	3.60E-18	pfam
	GJ5E19T3.1	GjbHLH24.4	MN385911	63	109	61	109	PF00010.26	HLH	4	53	53	53.29	2.00E-14	1.10E-18	pfam
	GJ1342E0T2.1	GjbHLH24.5	MN385912	136	182	133	182	PF00010.26	HLH	4	53	53	54.04	1.10E-14	6.40E-19	pfam
	GJ20E21T3.1	GjbHLH24.6	MN385913	136	182	133	182	PF00010.26	HLH	4	53	53	54.04	1.10E-14	6.40E-19	pfam

27	GJ884E0T0.1	GjbHLH27.1	MN385914	310	356	306	356	PF00010.26	HLH	5	53	53	39.72	3.40E-10	1.90E-14	pfam
	GJ118P6T0.1	GjbHLH27.2	MN385915	368	413	364	413	PF00010.26	HLH	6	53	53	38.94	6.00E-10	3.30E-14	pfam
	GJ18P5T14.1	GjbHLH27.3	MN385916	406	451	402	451	PF00010.26	HLH	6	53	53	38.44	8.50E-10	4.80E-14	pfam
	GJ97P1T33.1	GjbHLH27.4	MN385917	305	350	301	350	PF00010.26	HLH	6	53	53	36.07	4.70E-09	2.60E-13	pfam
	GJ143P6T1.1	GjbHLH27.5	MN385918	215	260	211	260	PF00010.26	HLH	6	53	53	41.24	1.10E-10	6.40E-15	pfam
	GJ251P2T1.1	GjbHLH27.6	MN385919	215	260	211	260	PF00010.26	HLH	6	53	53	41.24	1.10E-10	6.40E-15	pfam
	GJ1E25T3.1	GjbHLH26.1	MN385920	158	202	155	203	PF00010.26	HLH	5	52	53	31.85	9.80E-08	5.50E-12	pfam
	GJ9E28T2.1	GjbHLH26.2	MN385921	153	197	150	198	PF00010.26	HLH	5	52	53	32	8.80E-08	4.90E-12	pfam
25	GJ17E23T2.1	GjbHLH25.1	MN385922	171	218	169	218	PF00010.26	HLH	4	53	53	25.78	7.70E-06	4.30E-10	pfam
	GJ53E12T1.1	GjbHLH25.2	MN385923	158	205	156	205	PF00010.26	HLH	4	53	53	25.32	1.10E-05	6.00E-10	pfam
	GJ2P16T67.1	GjbHLH25.3	MN385924	115	162	113	162	PF00010.26	HLH	4	53	53	28.74	9.20E-07	5.10E-11	pfam
	GJ8E40T6.1	GjbHLH25.4	MN385925	185	232	183	232	PF00010.26	HLH	4	53	53	28.38	1.20E-06	6.60E-11	pfam
	GJ16E17T3.1	GjbHLH25.5	MN385926	236	284	234	284	PF00010.26	HLH	3	53	53	27.39	2.40E-06	1.30E-10	pfam
	GJ1E43T6.1	GjbHLH25.6	MN385927	371	418	369	418	PF00010.26	HLH	4	53	53	27.66	2.00E-06	1.10E-10	pfam
	GJ14E34T2.1	GjbHLH25.7	MN385928	203	250	201	250	PF00010.26	HLH	4	53	53	27.62	2.00E-06	1.10E-10	pfam
	GJ40P17T10.1	GjbHLH25.8	MN385929	200	247	198	247	PF00010.26	HLH	4	53	53	27.66	2.00E-06	1.10E-10	pfam
	GJ10E19T0.1	GjbHLH25.9	MN385930	39	86	37	86	PF00010.26	HLH	4	53	53	37.56	1.60E-09	9.00E-14	pfam
	GJ1258E0T0.1	GjbHLH25.10	MN385931	56	103	54	103	PF00010.26	HLH	4	53	53	35.46	7.30E-09	4.10E-13	pfam
	GJ205E3T0.1	GjbHLH25.11	MN385932	56	103	54	103	PF00010.26	HLH	4	53	53	35.46	7.30E-09	4.10E-13	pfam
23	GJ49E15T3.1	GjbHLH23.1	MN385933	55	148	47	148	PF14215.6	bHLH-MYC_N	96	175	175	55.84	6.50E-15	3.60E-19	pfam
	GJ15E5T0.1	GjbHLH23.2	MN385934	55	148	47	148	PF14215.6	bHLH-MYC_N	96	175	175	55.84	6.50E-15	3.60E-19	pfam
	GJ1E28T7.1	GjbHLH23.3	MN385935	55	148	47	148	PF14215.6	bHLH-MYC_N	96	175	175	55.84	6.50E-15	3.60E-19	pfam
	GJ94E3T0.1	GjbHLH23.4	MN385936	5	174	5	175	PF14215.6	bHLH-MYC_N	1	174	175	114.95	4.60E-33	2.60E-37	pfam
16	GJ5P41T29.1	GjbHLH16.1	MN385937	22	60	18	60	PF00010.26	HLH	18	53	53	21.3	0.00019	1.10E-08	pfam
33	GJ40E18T3.1	GjbHLH33.1	MN385938					cd00083	HLH	358	388			2.07E-03		BLASTP
	GJ317P0T53.1	GjbHLH33.2	MN385939	390	437	388	437	PF00010.26	HLH	4	53	53	28.25	1.30E-06	7.30E-11	pfam

Table S4. Analysis of the structural features and conserved motifs of *bHLH* genes in *G. jasminoides*.

subfamily	sequen ID	amino acids length(aa)	Mw（Da）	PI	cDNA length (bp)	gene length （bp）	exon number	motif	motif number
3	GjbHLH3.1	141	15895.57	6.74	666	1056	2	11,1,2,8,4	5
	GjbHLH3.2	222	24675.47	7.10	699	890	3	11,1,2,8,4,13	6
	GjbHLH3.3	217	24401.84	6.24	654	1271	3	11,1,2,8,4,13	6
	GjbHLH3.4	183	20616.76	7.71	552	1339	2	11,1,2,8,4	5
	GjbHLH3.5	344	37294.41	5.62	1035	3861	3	11,1,2,8,4,13	6
	GjbHLH3.6	332	35852.63	5.85	999	2297	3	11,1,2,8,4,13	6
	GjbHLH3.7	340	37446.48	5.21	1020	1502	3	11,1,2,8,4,13	6
	GjbHLH3.8	245	27203.16	9.14	738	1082	2	11,1,2,8,4	5
	GjbHLH3.9	309	34122.03	7.57	933	1365	4	11,1,2,8,4	5
	GjbHLH3.10	216	24355.37	8.69	648	2143	5	11,1,2	3
5	GjbHLH5.1	289	32210.98	9.93	870	2140	4	1,2,6	3
2	GjbHLH2.1	497	55308.45	6.14	1581	1581	1	12,7,16,1,2,6	6
	GjbHLH2.2	561	60061.91	5.14	1791	1791	1	12,7,1,2,6	5
	GjbHLH2.3	617	67311.87	5.79	1854	2004	4	12,7,16,1,2,6	6
	GjbHLH2.4	490	54331.00	5.72	1473	2430	3	12,7,16,1,2,6	6
	GjbHLH2.5	608	66645.79	6.36	1827	2671	3	12,7,16,1,2,6	6
1	GjbHLH1.1	280	30656.74	6.42	843	2571	5	1,2,6	3
	GjbHLH1.2	190	20951.42	5.33	573	770	2	1,2	2
	GjbHLH1.3	322	36184.89	4.82	969	1826	6	1,2,6	3
	GjbHLH1.4	236	26046.58	5.78	711	711	1	15,1	2
	GjbHLH1.5	129	14853.64	4.81	390	572	2	1,2，	2
	GjbHLH1.6	298	33509.04	4.82	897	1607	4	1,2,6	3
	GjbHLH1.7	233	26574.00	5.04	702	4722	7	1,2,4	3
	GjbHLH1.8	193	21722.01	8.37	348	4108	5	1,2,4	3
	GjbHLH1.9	193	21722.01	8.37	348	3904	5	1,2,4	3
7	GjbHLH7.1	495	54848.74	7.16	1485	2331	7	1,2	2
	GjbHLH7.2	182	21196.60	9.32	549	668	2	1,2	2
4	GjbHLH4.1	234	25790.27	7.69	705	3709	7	1,2,21	3
	GjbHLH4.2	236	26305.56	6.15	711	3820	7	1,2,21	3
	GjbHLH4.3	238	26830.45	5.46	717	4216	7	1,2,21	3
	GjbHLH4.4	239	26718.98	7.14	720	2245	6	1,2,21	3
	GjbHLH4.5	248	27347.60	5.27	747	3536	6	1,2,21	3
	GjbHLH4.6	338	37540.60	6.14	1017	4728	7	1,2,21	3
9	GjbHLH9.1	349	38679.34	8.85	1050	1180	2	1,2,6	3
14	GjbHLH14.1	252	28625.68	8.22	759	855	2	1,2，	2
	GjbHLH14.2	333	36210.62	9.48	1002	2769	8	1,2	2
11	GjbHLH11.1	131	14472.98	6.91	396	396	1	1	1
12	GjbHLH12.1	221	25125.27	8.86	666	1921	2	1,2	2
10	GjbHLH10.1	411	46308.09	5.18	1236	2382	6	1,2,4	3
	GjbHLH10.2	166	18803.22	9.71	501	1340	3	1,2,6	3

	GjbHLH10.3	191	21381.37	7.85	576	926	4	1,2,4	3
	GjbHLH10.4	221	25345.89	9.32	666	1223	3	1,2,4	3
	GjbHLH10.5	217	24284.47	6.44	654	1353	4	1,2,4	3
	GjbHLH13.1	93	10481.09	9.23	279	409	2	1,2	2
	GjbHLH13.2	201	22362.73	8.97	603	1114	2	1,2,6	3
13	GjbHLH13.3	232	25607.32	7.78	699	1862	4	1,2,6	3
	GjbHLH13.4	259	28737.82	8.65	813	1403	2	1,2,6	3
	GjbHLH13.5	245	27683.13	5.54	738	1842	4	1,2,6	3
	GjbHLH13.6	308	33781.92	5.58	924	2045	3	1,2,6	3
	GjbHLH15.1	354	38842.73	8.35	1065	4858	8	1,2,9	3
	GjbHLH15.2	428	48101.93	8.55	1284	3318	7	1,2,9	3
	GjbHLH15.3	347	38051.41	6.39	1044	3866	11	1,2,9	3
15	GjbHLH15.4	280	30092.43	8.67	843	3360	8	1,2,9	3
	GjbHLH15.5	468	50952.12	5.36	1404	2508	8	1,2,9	3
	GjbHLH15.6	472	51344.67	6.16	1416	2352	7	1,2,9	3
	GjbHLH15.7	490	54052.50	7.07	1473	4017	9	1,2,9	3
32	GjbHLH32.1	292	31941.51	5.53	879	4474	9	1,2,9	3
	GjbHLH31.1	132	14994.33	10.42	399	399	1	10,1,2	3
	GjbHLH31.2	136	15542.19	10.05	411	928	3	10,1,2	3
31	GjbHLH31.3	448	49184.61	5.62	1347	2419	3	10,1,2	3
	GjbHLH31.4	144	15947.58	10.51	435	435	1	10,1,2	3
	GjbHLH31.5	127	14245.40	6.45	384	384	1	1,2	2
30	GjbHLH30.1	335	37110.86	7.00	1035	1035	1	1,2	3
	GjbHLH24.1	524	57718.04	6.78	1575	4071	9	1,2	2
	GjbHLH24.2	520	57179.29	5.85	1680	5656	8	1,2	2
24	GjbHLH24.3	403	44702.02	8.93	1257	3431	5	1,2	2
	GjbHLH24.4	228	24968.40	7.95	687	3005	6	1,2	2
	GjbHLH24.5	317	33812.39	5.59	954	3574	8	18,1,2,20	4
	GjbHLH24.6	317	33812.39	5.59	954	3571	8	18,1,2,20	4
	GjbHLH27.1	383	41515.15	7.63	1149	2347	7	15,15,17,3,1,2	6
	GjbHLH27.2	428	46586.08	5.41	1323	2529	6	15,15,17,3,1,2	6
27	GjbHLH27.3	434	47428.24	7.10	1437	2467	6	15,15,17,3,1,2	6
	GjbHLH27.4	358	39035.36	8.98	1140	11590	6	15,15,3,1,2	5
	GjbHLH27.5	256	27384.76	5.72	819	2939	4	15,15,3,1,2	5
	GjbHLH27.6	**256**	27355.72	5.99	819	2939	4	15,15,3,1,2	5
26	GjbHLH26.1	309	32715.10	6.00	930	4046	8	3,1,2,9	4
	GjbHLH26.2	304	32993.21	5.05	915	5167	9	3,3,1,2,9	5
	GjbHLH25.1	243	27389.88	8.41	732	1883	7	3,1,2,	3
	GjbHLH25.2	266	29699.24	6.42	801	2017	8	3,1,2,	3
	GjbHLH25.3	177	19809.16	9.28	534	874	3	3,1,2,	3
25	GjbHLH25.4	351	39574.13	6.23	1056	2617	8	3,1,2,	3
	GjbHLH25.5	442	48354.01	6.71	1326	2606	8	3,1,2,	3
	GjbHLH25.6	439	47218.79	8.31	1320	2853	7	3,1,2,	**3**
	GjbHLH25.7	375	42097.51	8.18	1128	3379	9	3,1,2,	3

	GjbHLH25.8	364	40462.58	5.86	1131	4138	6	3,1,2,	3
	GjbHLH25.9	89	9904.32	9.25	267	1651	4	3,1,	2
	GjbHLH25.10	173	19413.00	6.52	522	3278	6	3,1,2,	3
	GjbHLH25.11	173	19341.82	6.53	522	3278	6	3,1,2,	3
23	GjbHLH23.1	826	91898.49	5.11	2481	5112	12	12,14,1,22,5	5
	GjbHLH23.2	726	80829.11	5.60	2181	6975	12	12,14,1,22,5	5
	GjbHLH23.3	693	77013.8	5.33	2079	6479	11	12,14,1,22,5	5
	GjbHLH23.4	966	104750.2	5.9	2901	6330	12	12,14,1,22,5	5
16	GjbHLH16.1	92	10351.89	9.09	279	673	2	2	1
33	GjbHLH33.1	422	45286.34	8.39	1266	2527	5	15,15,19,3,1	5
	GjbHLH33.2	446	47456.87	6.63	1338	1137	3	3,1	2

Table S5. Conservative motifs predicted by MEME

Motif No.	E-value	Sequence
MOTIF-1	3.3e-1194	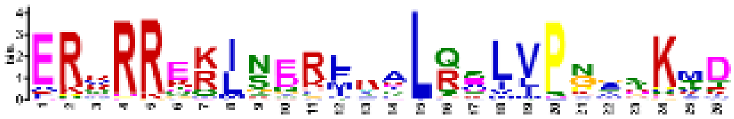<br>ERRRRE[KR][IL][NS][ED]R[LF]KAL[QR]SLVP[NG]XXKMD
MOTIF-2	9.3e-899	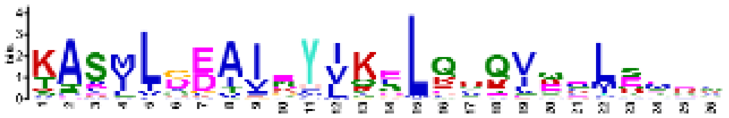<br>[KT]AS[MVI]L[DG][ED]AIEY[IVL]K[EF]LQXQ[VI]QXL[SE]XXX
MOTIF-3	1.5e-259	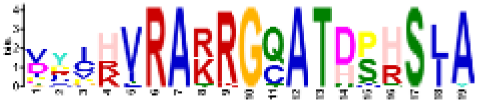<br>[VD]Y[IC][HKR]VRA[RK]RG[QC]AT[DH][PS][HR]S[LI]A
MOTIF-4	6.4e-202	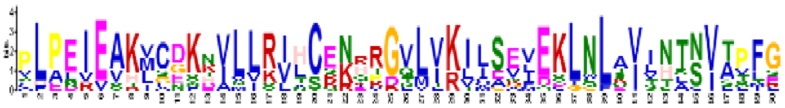<br>PL[PA]EIE[AV]K[LMV]C[DEG]K[NK][VI]LL[RK][IV][HL][CS]E[NK][HR]RG[VQ]L[VI][KR][IL][LI]S[EV][VIL]EKLNLA[VI][IL][NH]T[NS][VI][TA]PFG
MOTIF-5	4.3e-095	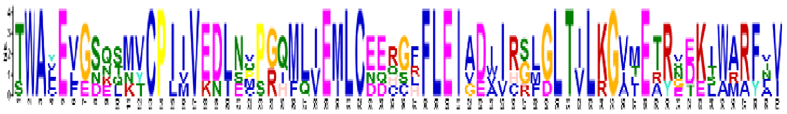<br>[TS]WA[CFLVY]E[VFL][GE][SDN][QEKN][SLQT][MKN][VTY]CP[IL][IMV]V[EK][DN][LI][NES][CMPQV][PS][GR][QHI][MF][LQ][IV]E[MI]LC[EDN][EDQ][RCHQ][GCS][FHLR]FLEI[AGV][DE][IAVW][IV][RCH][GSR][LFM][GD]LT[IV]L[KR]G[VAI][MILT]E[TAR][RY][EGNSV][DET][KE][ILST][WA][AMR][RA][FY][AFNTV][VI]
MOTIF-6	7.4e-091	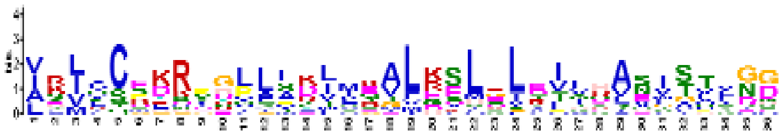<br>[VIA][KR][LIV]CCE[KD]RPG[LP]L[LS][DK][LI]MXALK[SE]LXLE[IV]LHASI[ST]TX[GN][GD]
MOTIF-7	5.8e-080	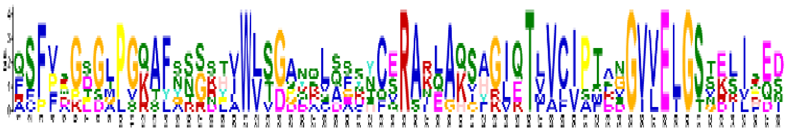<br>[QF]SF[VFP]XG[DS]GLPG[KQ]AF[NS][SN][GS][KS][HT][VI]W[LV][ST][GD]ANQL[AQS][FS]S[NY]C[ES]RA[KR][LEQ]A[KQ]S[AH]G[IL][QE]T[LV]VC[IV]PTA[GN]GV[VL]ELGS[ST][EK][LS]IX[EQ][DS]

MOTIF-8	5.6e-070	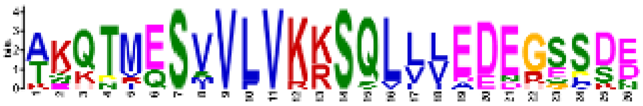 [AT]K[QK]TM[EQ]SVVLVK[KR]SQL[LV][LV]E[DE]EG[SE]S[DS][ED]
MOTIF-9	1.5e-066	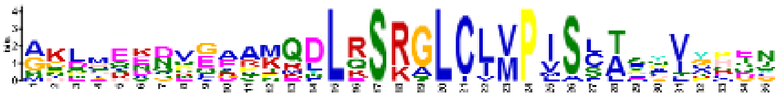 [AG]KLME[EK][DN]V[EG][AE]A[KM]QDL[RQ]S[RK]GLC[LI][VM]P[IV]S[CLS][TA]SX[VI]Y[HP]EN
MOTIF-10	2.8e-060	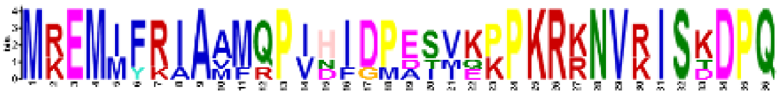 M[RK]EM[IM][FY][RK][IA]A[AMV][MF][QR]P[IV][HDN][IF][DG][PM][EAD][SIT][VIM][KEQ][PK]PKR[KR]NV[RK]IS[KDT]DPQ
MOTIF-11	1.5e-053	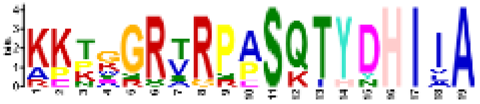 [KA][KP][TKP][GK]GR[TV]RP[AP]S[QK]TYDHIIA
MOTIF-12	1.9e-050	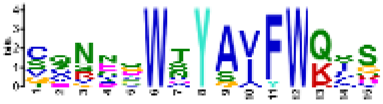 [CS]QN[EN]XW[TS]YA[VI]FW[QK][IS]S
MOTIF-13	2.1e-040	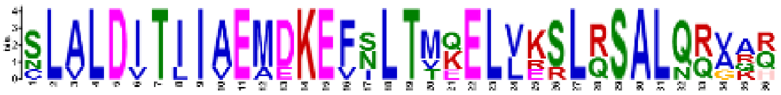 [SCN]L[AV]LD[IV]T[IL]I[AV]E[MA][DE]KE[FV][NSI]LT[MTV][KQE]EL[VL][KER][SR]L[RQ]SAL[QN][RQ][AVG][AKQR][QRH]
MOTIF-14	1.6e-036	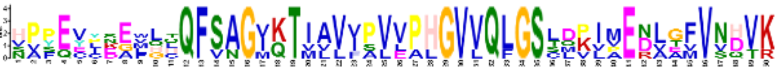 [HNSV][PAV][PFSY][EQ][VCEY][CILPY][DEKNS][EAG][FLMVW][LGHQ][CHLQT]QF[SV][AN]G[IVM][KQ]T[IMV][ALV][VL][IVF][PAS][VL][VEL][PA][HL]GV[VL]Q[LF]GS[LCST][DLMQ][KPV][ILV][MAPV]E[DNR][LAV][GAET][FMV]V[NSV][DHQ][VT][KR]
MOTIF-15	3.6e-032	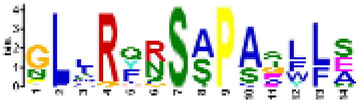 GLLR[QFY][RN]S[AS]P[AS]S[LF][LF][SEA]
MOTIF-16	5.5e-030	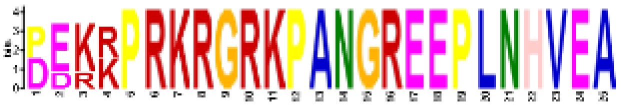 [DP][ED][KR][KR]PRKRGRKPANGREEPLNHVEA

MOTIF-17	1.9e-029	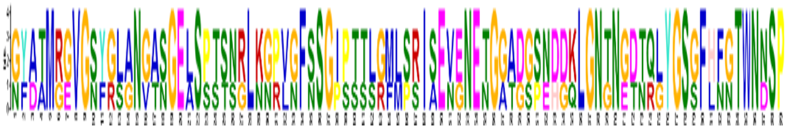<br>[GN][YF][AD][TA]M[RG][GE]VG[SN][YF][GR][LS][AG]N[GV][AT][SN]GE[LA]S[PS][TS][ST][NS][RG]L[KN][GN][PR][VL][GN]F[SN]SG[IP][PS][TS][TS][LS][GR][MF][LM][SP][RS]I[SA]E[VN][EG]NE[TN]G[GA][AT][DG][GS][SP][NE][DH][DG][KQ]LGN[TG]N[GE][DT][TN][QR][LG]YGS[GS]F[HL][FN][GN]TWN[ND]SP
MOTIF-18	8.8e-043	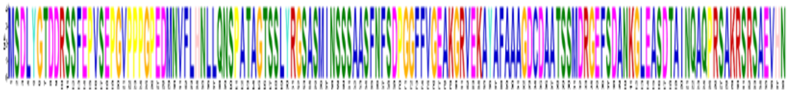<br>MSDLYGTDDRSSFEPVSEPGVPPPGPEDMNVFLHNLLQNSPATAGTSSLYRGSASMINSSSAASFNFSDPGGFFVGEAKGRVEKAYAFAAAGDCDAATSSMDRGEFSDANKGLEASDTAINQAQPRSAKRSRSAEVHN
MOTIF-19	3.2e-033	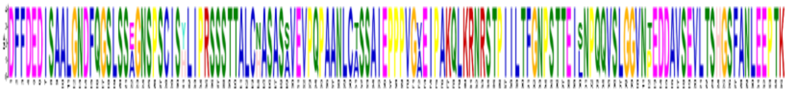<br>DFFDEDISAALGNDFQGSLSS[AE]GNSPSCIS[HY]LIPRSSSTTALC[HN]ASAS[AS]VEVPQPAANLC[AT]SSAIEPPPVG[AV]EIPAKQLKRNRSTPIILTFGNPSTTEI[LS]NPQQVSLGGVN[PT]EDDAVSEVLTSHGSFANLEEPTK
MOTIF-20	E= 2.7e-031	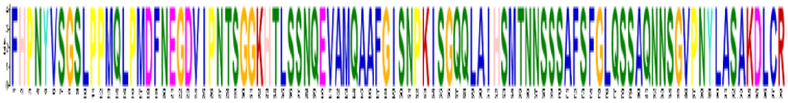<br>FHPNYVSGSLPPMQLPMDFNEGDVIPNTSGGKHTLSSNQEVAMQAAFGISNPKISGQQLAIHSMTNNSSSAFSFGLQSSAQNNSGVPNYLASAKDLCR
MOTIF-21	7.2e-029	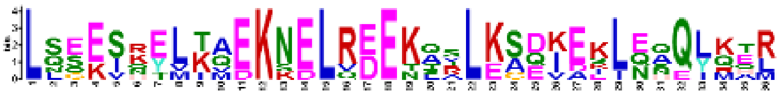<br>L[SQ][ES][EK][SI][KR][EY]L[KT][AQ]EKNELR[ED]EK[AQ]SL[KE][SA][DEQ][IK]EKL[ENQ][AQ]Q[LY][KQ][ET][RL]
MOTIF-22	1.9e-026	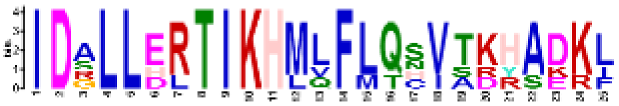<br>ID[AGRS]LL[EDH][RL]TIKH[ML][LQV]F[LM][QT][SCHN][VI][TAS][KDR][HRY][AS][DEK][KR][LFI]

Table S6. The FPKM values of 95 *GjbHLH* genes in different organs.

subfamily	sequen ID	Root	Stem	Leaf	Young Fruit	Green Fruit	Red Fruit	CCD4a-co-expression*	ALDH-co-expression*	UGT74F8-co-expression*	UGT94E13-co-expression*
	GjbHLH3.1	15.81	2.11	0.30	1.02	0.00	0.08	-0.39	-0.37	-0.41	-0.39
	GjbHLH3.2	0.42	0.00	1.18	0.16	0.04	0.00	-0.47	-0.42	-0.48	-0.47
	GjbHLH3.3	0.55	0.00	1.25	0.77	0.00	0.00	-0.64	-0.57	-0.63	-0.63
	GjbHLH3.4	12.32	0.18	0.63	0.37	0.07	0.25	-0.34	-0.30	-0.35	-0.33
	GjbHLH3.5	0.22	0.63	0.38	0.07	0.00	0.00	-0.67	-0.71	-0.69	-0.66
3	GjbHLH3.6	0.14	0.61	0.16	0.82	0.29	0.08	-0.45	-0.49	-0.38	-0.46
	GjbHLH3.7	0.76	0.03	2.52	0.97	0.05	0.00	-0.55	-0.49	-0.55	-0.55
	GjbHLH3.8	0.00	0.00	0.22	0.00	0.00	0.00	-0.32	-0.28	-0.33	-0.31
	GjbHLH3.9	0.00	0.60	0.10	0.00	0.00	0.00	-0.38	-0.45	-0.40	-0.37
	GjbHLH3.10	324.70	60.69	4.94	7.50	1.11	0.96	-0.39	-0.38	-0.41	-0.39
5	GjbHLH5.1	0.56	0.69	0.01	2.79	5.61	4.10	0.87	0.83	0.91	0.84
	GjbHLH2.1	24.04	0.25	0.45	1.39	2.61	0.07	-0.29	-0.27	-0.29	-0.29
	GjbHLH2.2	150.83	106.02	85.40	146.79	56.85	61.78	-0.79	-0.76	-0.78	-0.78
2	GjbHLH2.3	156.37	252.45	497.05	255.91	39.47	29.09	-0.76	-0.73	-0.76	-0.76
	GjbHLH2.4	306.06	91.07	435.98	303.36	28.76	31.53	-0.76	-0.70	-0.76	-0.76
	GjbHLH2.5	336.32	223.92	403.18	230.40	13.68	13.74	-0.91	-0.87	-0.92	-0.90
1	GjbHLH1.1	4.34	3.10	5.03	8.03	4.06	3.67	-0.37	-0.33	-0.32	-0.38

	GjbHLH1.2	3.12	49.58	43.60	15.32	5.50	9.78	-0.51	-0.54	-0.54	-0.50
	GjbHLH1.3	33.65	0.43	0.02	3.02	3.53	7.22	-0.15	-0.11	-0.17	-0.14
	GjbHLH1.4	0.52	4.67	1.03	1.50	0.45	0.36	-0.48	-0.54	-0.48	-0.48
	GjbHLH1.5	0.39	0.00	0.00	0.21	1.56	0.00	0.48	0.38	0.59	0.41
	GjbHLH1.6	0.23	0.22	0.05	0.01	0.00	0.02	-0.56	-0.58	-0.59	-0.55
	GjbHLH1.7	786.99	171.93	690.96	434.99	13.91	16.29	-0.77	-0.71	-0.78	-0.77
	GjbHLH1.8	0.00	0.00	0.00	0.00	0.00	1.78	0.68	0.76	0.58	0.74
	GjbHLH1.9	786.99	171.93	690.96	434.99	13.91	16.29	-0.77	-0.71	-0.78	-0.77
	GjbHLH7.1	1.36	0.45	1.09	1.89	0.14	0.94	-0.51	-0.40	-0.54	-0.47
7	GjbHLH7.2	98.05	90.57	561.99	232.70	0.04	0.06	-0.59	-0.55	-0.60	-0.59
	GjbHLH4.1	253.94	104.32	217.23	142.40	95.68	89.00	-0.65	-0.59	-0.65	-0.65
	GjbHLH4.2	7.97	10.46	26.68	6.03	3.07	3.68	-0.55	-0.52	-0.57	-0.55
4	GjbHLH4.3	34.29	37.11	55.62	32.07	42.43	32.42	-0.16	-0.18	-0.13	-0.19
	GjbHLH4.4	14.19	17.54	12.06	11.68	1.98	4.04	-0.92	-0.92	-0.94	-0.91
	GjbHLH4.5	57.04	73.09	84.39	66.27	18.73	17.17	-0.95	-0.93	-0.95	-0.94
	GjbHLH4.6	9.50	10.22	12.31	10.44	9.18	8.98	-0.65	-0.62	-0.65	-0.65
9	GjbHLH9.1	0.90	0.17	0.02	0.64	0.12	0.09	-0.47	-0.44	-0.45	-0.47
14	GjbHLH14.1	0.33	0.44	0.37	0.37	5.84	4.86	0.98	0.95	1.00	0.96
	GjbHLH14.2	16.71	9.30	7.08	20.86	10.77	17.92	0.11	0.18	0.09	0.14
11	GjbHLH11.1	0.10	0.00	0.00	1.65	0.11	0.80	0.04	0.10	0.04	0.06
12	GjbHLH12.1	0.00	0.00	0.04	0.00	0.00	0.00	-0.32	-0.28	-0.33	-0.31
10	GjbHLH10.1	0.00	0.00	0.93	0.59	0.08	0.10	-0.39	-0.33	-0.38	-0.39

	GjbHLH10.2	0.09	0.05	0.65	0.22	0.19	0.04	-0.32	-0.29	-0.30	-0.33
	GjbHLH10.3	1.39	1.12	0.00	0.00	0.14	0.00	-0.46	-0.49	-0.47	-0.46
	GjbHLH10.4	13.00	0.74	0.50	0.73	0.00	0.00	-0.37	-0.34	-0.38	-0.37
	GjbHLH10.5	8.65	10.46	0.07	3.35	1.22	0.17	-0.57	-0.62	-0.57	-0.57
	GjbHLH13.1	2.13	0.45	0.13	0.04	0.00	0.00	-0.42	-0.41	-0.44	-0.42
	GjbHLH13.2	0.00	0.00	0.27	0.00	2.63	0.96	0.82	0.75	0.89	0.77
13	GjbHLH13.3	3.35	4.18	0.16	1.16	1.30	1.24	-0.32	-0.37	-0.33	-0.32
	GjbHLH13.4	6.31	3.25	1.18	3.85	9.32	15.44	0.90	0.92	0.85	0.92
	GjbHLH13.5	2.60	4.71	1.47	2.69	1.92	1.06	-0.56	-0.64	-0.54	-0.58
	GjbHLH13.6	1.29	3.63	2.08	0.57	1.19	2.03	-0.12	-0.16	-0.19	-0.10
	GjbHLH15.1	3.91	4.98	3.70	1.71	1.89	1.51	-0.68	-0.72	-0.70	-0.68
	GjbHLH15.2	1.58	11.28	0.52	0.47	0.59	1.67	-0.28	-0.34	-0.31	-0.27
	GjbHLH15.3	43.61	39.44	24.86	24.59	34.84	28.86	-0.10	-0.16	-0.09	-0.12
15	GjbHLH15.4	3.38	3.15	3.46	3.47	12.50	5.55	0.76	0.67	0.84	0.70
	GjbHLH15.5	45.25	15.56	46.41	49.45	3.24	2.79	-0.83	-0.77	-0.82	-0.83
	GjbHLH15.6	5.37	4.67	1.88	6.64	4.71	3.93	-0.11	-0.14	-0.06	-0.12
	GjbHLH15.7	0.42	1.76	0.35	0.00	4.51	1.94	0.76	0.66	0.82	0.72
32	GjbHLH32.1	3.84	6.73	5.03	4.28	2.50	2.41	-0.80	-0.83	-0.80	-0.79
	GjbHLH31.1	0.00	0.00	0.00	0.00	0.50	0.42	0.98	0.95	1.00	0.97
	GjbHLH31.2	8.64	0.45	0.12	0.18	1.31	0.42	-0.23	-0.22	-0.23	-0.24
31	GjbHLH31.3	0.09	0.00	6.49	0.91	0.14	0.00	-0.36	-0.32	-0.37	-0.36
	GjbHLH31.4	0.00	0.00	0.00	0.35	0.22	0.36	0.61	0.64	0.61	0.62
	GjbHLH31.5	4.13	2.02	0.19	0.00	0.00	0.00	-0.48	-0.48	-0.50	-0.48
30	GjbHLH30.1	0.00	0.06	0.03	0.08	0.63	0.27	0.84	0.76	0.91	0.80
24	GjbHLH24.1	2.24	27.25	44.93	12.05	5.41	7.55	-0.47	-0.47	-0.49	-0.47
	GjbHLH24.2	0.25	2.55	7.23	2.51	2.90	2.47	-0.11	-0.09	-0.11	-0.11

	GjbHLH24.3	0.02	0.46	4.37	0.28	0.24	0.25	-0.32	-0.28	-0.33	-0.32
	GjbHLH24.4	12.14	10.62	2.78	10.44	3.09	1.35	-0.73	-0.75	-0.71	-0.73
	GjbHLH24.5	2.83	0.93	3.07	0.66	0.27	0.47	-0.62	-0.56	-0.65	-0.61
	GjbHLH24.6	19.57	8.50	10.80	4.79	3.21	0.98	-0.69	-0.67	-0.69	-0.69
	GjbHLH27.1	0.00	0.61	1.28	1.92	0.89	0.00	-0.37	-0.39	-0.29	-0.40
	GjbHLH27.2	6.64	14.95	35.65	19.88	48.56	47.23	0.84	0.83	0.84	0.83
27	GjbHLH27.3	323.00	221.68	318.49	267.46	6.40	7.78	-0.97	-0.93	-0.97	-0.96
	GjbHLH27.4	1.00	8.41	3.82	5.47	4.99	6.07	0.19	0.13	0.17	0.20
	GjbHLH27.5	13.49	18.75	19.61	15.52	4.30	3.65	-0.95	-0.94	-0.95	-0.94
	GjbHLH27.6	4.03	4.81	5.74	5.20	4.74	2.53	-0.64	-0.70	-0.56	-0.69
	GjbHLH26.1	25.16	57.75	46.95	37.85	43.92	44.00	0.10	0.03	0.08	0.10
26	GjbHLH26.2	20.28	42.58	103.08	53.75	69.12	96.65	0.47	0.52	0.43	0.49
	GjbHLH25.1	1.35	5.79	0.42	0.40	0.06	0.36	-0.41	-0.48	-0.44	-0.41
	GjbHLH25.2	0.83	2.65	0.14	1.64	3.50	5.02	0.86	0.84	0.83	0.87
	GjbHLH25.3	0.41	0.91	8.22	0.64	2.90	1.17	-0.10	-0.09	-0.09	-0.12
	GjbHLH25.4	1.32	0.16	0.60	0.36	0.00	0.00	-0.62	-0.57	-0.63	-0.62
	GjbHLH25.5	2.29	7.34	9.26	9.59	1.45	1.00	-0.76	-0.75	-0.74	-0.76
25	GjbHLH25.6	5.53	3.02	1.71	5.99	3.05	5.13	0.03	0.10	0.01	0.06
	GjbHLH25.7	0.05	13.40	9.20	12.88	5.45	5.66	-0.34	-0.37	-0.32	-0.33
	GjbHLH25.8	0.24	1.37	0.00	0.00	0.46	0.93	0.29	0.24	0.24	0.31
	GjbHLH25.9	3.49	8.67	8.49	28.88	5.80	4.45	-0.40	-0.38	-0.35	-0.40
	GjbHLH25.10	0.44	3.83	7.46	5.44	4.08	2.81	-0.20	-0.20	-0.16	-0.21
	GjbHLH25.11	1.13	4.44	9.84	7.40	3.18	2.75	-0.44	-0.41	-0.42	-0.44
23	GjbHLH23.1	1.09	3.17	0.06	2.27	4.55	5.11	0.84	0.80	0.84	0.84

	GjbHLH23.2	13.53	8.39	9.16	18.93	35.51	28.71	0.90	0.88	0.94	0.88
	GjbHLH23.3	15.32	10.28	9.48	14.48	11.18	7.57	-0.55	-0.56	-0.48	-0.57
	GjbHLH23.4	7.08	35.33	7.05	17.52	26.14	21.53	0.32	0.22	0.34	0.31
16	GjbHLH16.1	0.00	0.00	0.00	0.00	0.00	0.00				
33	GjbHLH33.1	0.05	0.55	2.26	1.99	1.12	0.94	-0.12	-0.08	-0.09	-0.12
	GjbHLH33.2	12.11	7.90	5.24	9.95	1.55	1.64	-0.85	-0.83	-0.84	-0.84
	GjCCD4a*	0.00	0.00	0.00	0.05	1403.67	1572.05				
	GjALDH2C3 (ALDH12)*	57.51	7.85	62.23	56.67	415.09	552.13				
	UGT74F8*	14.28	8.04	7.81	86.56	1315.29	1183.66				
	UGT94E13 (UGT60)*	0.59	0.43	0.11	0.68	177.26	225.63				

Asterisks (*) represent the co-expression of four genes (*GjALDH2C3*, *GjUGT94E13*, *GjUGT74F8* and *CjCCD4a*) in crocin biosynthesis.

Table S7. The qPCR analysis for candidate *GjbHLH* genes.

sequence ID	organ	Ct-value					
		treatment 1	treatment 2	treatment 3	actin 1	actin 2	actin 3
GjbHLH3.10	Root	26.47	26.58	26.53	22.29	22.28	22.35
	Stem	27.16	27.06	27.15	21.28	21.27	21.25
	Leaf	31.13	31.21	31.02	22.28	22.30	22.33
	Young Fruit	34.04	34.49	33.99	19.13	19.12	19.17
	Red Fruit	31.19	31.52	31.35	20.20	20.20	20.16
GjbHLH2.2	Root	22.96	23.03	23.02	22.83	22.87	22.70
	Stem	22.50	22.39	22.44	21.38	21.54	21.52
	Leaf	22.41	22.46	22.72	22.70	22.84	22.82
	Young Fruit	23.24	23.24	23.14	21.42	21.33	21.41
	Red Fruit	26.36	26.23	26.51	22.37	22.28	22.48
GjbHLH2.3	Root	22.96	23.07	23.02	22.83	22.87	22.70
	Stem	20.69	20.67	20.65	21.38	21.54	21.52
	Leaf	20.20	20.25	20.33	22.70	22.84	22.82
	Young Fruit	21.21	21.05	21.06	21.42	21.33	21.41
	Red Fruit	23.77	23.80	23.72	22.37	22.28	22.48
GjbHLH2.4	Root	23.14	23.23	23.16	22.83	22.87	22.70
	Stem	23.45	23.33	23.27	21.38	21.54	21.52
	Leaf	22.22	22.48	22.47	22.70	22.84	22.82
	Young Fruit	23.32	23.17	23.29	21.42	21.33	21.41
	Red Fruit	23.95	24.09	24.05	22.37	22.28	22.48
GjbHLH2.5	Root	22.30	22.37	22.43	22.83	22.87	22.70
	Stem	21.03	21.04	21.12	21.38	21.54	21.52
	Leaf	20.87	20.86	21.20	22.70	22.84	22.82
	Young Fruit	22.08	22.06	22.02	21.42	21.33	21.41
	Red Fruit	24.93	24.93	25.00	22.37	22.28	22.48
GjbHLH1.7	Root	21.68	21.76	21.86	22.83	22.87	22.70
	Stem	22.59	22.52	22.51	21.38	21.54	21.52
	Leaf	21.02	21.13	21.23	22.70	22.84	22.82
	Young Fruit	22.46	22.48	22.51	21.42	21.33	21.41
	Red Fruit	25.34	25.49	25.48	22.37	22.28	22.48
GjbHLH7.2	Root	25.90	25.93	25.92	22.83	22.87	22.70
	Stem	24.26	24.14	24.16	21.38	21.54	21.52
	Leaf	22.09	22.18	22.41	22.70	22.84	22.82
	Young Fruit	26.17	26.19	26.18	21.42	21.33	21.41
	Red Fruit	29.78	29.81	29.60	22.37	22.28	22.48
GjbHLH1.9	Root	29.53	29.38	29.20	22.83	22.87	22.70
	Stem	29.31	29.60	29.39	21.38	21.54	21.52
	Leaf	27.86	28.21	28.35	22.70	22.84	22.82
	Young Fruit	29.14	29.29	29.15	21.42	21.33	21.41
	Red Fruit	29.00	28.86	28.65	22.37	22.28	22.48
GjbHLH27.3	Root	21.37	21.37	21.53	22.83	22.87	22.70

	Stem	22.21	22.29	22.30	21.38	21.54	21.52
	Leaf	20.97	21.07	21.16	22.70	22.84	22.82
	Young Fruit	20.62	20.65	20.59	21.42	21.33	21.41
	Red Fruit	25.52	25.37	25.53	22.37	22.28	22.48

Table S8. The *cis*-acting elements of the promoter sequences of 6 key enzyme genes in crocin biosynthesis pathway.

Gene name		Factor Site Name	site
GJ3P18T16<br>GjBCH	E-box	EBOXBNNAPA	388 (+) CANNTG; 446 (+) CANNTG; 801 (+) CANNTG;<br>1020 (+) CANNTG; 1028 (+) CANNTG; 1288 (+) CANNTG;<br>1489 (+) CANNTG;<br>388 (-) CANNTG; 446 (-) CANNTG; 801 (-) CANNTG;<br>1020 (-) CANNTG; 1028 (-) CANNTG; 1288 (-) CANNTG;<br>1489 (-) CANNTG;
		MYCATRD22	446 (+) CACATG; 801 (-) CACATG;
GJ1P111T20<br>GjLCYB	E-box	EBOXBNNAPA	471 (+) CANNTG; 744 (+) CANNTG; 1205 (+) CANNTG;<br>471 (-) CANNTG; 744 (-) CANNTG; 1205 (-) CANNTG;
		MYCATRD22	744 (-) CACATG;
GJ6E31T6<br>GjALDH2C3	E-box	EBOXBNNAPA	117 (+) CANNTG; 882 (+) CANNTG; 1005 (+) CANNTG;<br>1220 (+) CANNTG;<br>117 (-) CANNTG; 882 (-) CANNTG; 1005 (-) CANNTG;<br>1220 (-) CANNTG;
		MYCATRD22	117 (+) CACATG;
	T/G-box	T/GBOXATPIN2	1208 (-) AACGTG; 1402 (+) AACGTG;
GJ3P78T69<br>GjUGT94E13	E-box	EBOXBNNAPA	625 (+) CANNTG; 945 (+) CANNTG; 971 (+) CANNTG;<br>1170 (+) CANNTG; 1368 (+) CANNTG;<br>625 (-) CANNTG; 945 (-) CANNTG; 971 (-) CANNTG;<br>1170 (-) CANNTG; 1368 (-) CANNTG;
	T/G-box	T/GBOXATPIN2	953 (-) AACGTG;
GJ1P46T104<br>GjUGT74E8	E-box	EBOXBNNAPA	165 (+) CANNTG; 171 (+) CANNTG; 232 (+) CANNTG;<br>509 (+) CANNTG; 578 (+) CANNTG; 737 (+) CANNTG;<br>848 (+) CANNTG; 1072 (+) CANNTG; 1178 (+) CANNTG;<br>165 (-) CANNTG; 171 (-) CANNTG; 232 (-) CANNTG;<br>509 (-) CANNTG; 578 (-) CANNTG; 737 (-) CANNTG;<br>848 (-) CANNTG; 1072 (-) CANNTG; 1178 (-) CANNTG;
		MYCATRD22	171 (+) CACATG;
	G-box	CACGTGMOTIF	165 (-) CACGTG; 165 (+) CACGTG;
GJ52E13T3<br>GjCCD4a	E-box	EBOXBNNAPA	70 (+) CANNTG; 365 (+) CANNTG; 669 (+) CANNTG;<br>1252 (+) CANNTG;<br>70 (-) CANNTG; 365 (-) CANNTG; 669 (-) CANNTG;<br>1252 (-) CANNTG;
	T/G-box	T/GBOXATPIN2	409 (-) AACGTG;

Table S9. The *cis*-acting elements of the promoter sequences of candidate *GjbHLH* genes.

Gene name		Foctor or site name	site
GJ458E2T0 GjbHLH1.7	E-box	EBOXBNNAPA	408 (+) CANNTG; 937 (+) CANNTG; 1130 (+) CANNTG; 1218 (+) CANNTG; 408 (-) CANNTG; 937 (-) CANNTG; 1130 (-) CANNTG; 1218 (-) CANNTG;
GJ458E1T0.1 GjbHLH1.9	E-box	EBOXBNNAPA	715 (+) CANNTG; 1007 (+) CANNTG; 1077 (+) CANNTG; 1085 (+) CANNTG; 1296 (+) CANNTG; 715 (-) CANNTG; 1007 (-) CANNTG; 1077 (-) CANNTG; 1085 (-) CANNTG; 1296 (-) CANNTG;
GJ4P54T24 GjbHLH2.2	E-box	EBOXBNNAPA	156 (+) CANNTG; 218 (+) CANNTG; 335 (+) CANNTG; 503 (+) CANNTG; 760 (+) CANNTG; 853 (+) CANNTG; 905 (+) CANNTG; 1018 (+) CANNTG; 1116 (+) CANNTG; 1129 (+) CANNTG; 1250 (+) CANNTG; 156 (-) CANNTG; 218 (-) CANNTG; 335 (-) CANNTG; 503 (-) CANNTG; 760 (-) CANNTG; 853 (-) CANNTG; 905 (-) CANNTG; 1018 (-) CANNTG; 1116 (-) CANNTG; 1129 (-) CANNTG; 1250 (-) CANNTG;
GJ115P5T25 GjbHLH2.3	E-box	EBOXBNNAPA	143 (+) CANNTG; 356 (+) CANNTG; 416 (+) CANNTG; 589 (+) CANNTG; 1156 (+) CANNTG; 1167 (+) CANNTG; 143 (-) CANNTG; 356 (-) CANNTG; 416 (-) CANNTG; 589 (-) CANNTG; 1156 (-) CANNTG; 1167 (-) CANNTG;
		MYCATRD22	1156 (-) CACATG; 1167 (+) CACATG;
GJ1E27T2 GjbHLH2.4	E-box	EBOXBNNAPA	54 (+) CANNTG; 310 (+) CANNTG; 594 (+) CANNTG; 613 (+) CANNTG; 758 (+) CANNTG; 1105 (+) CANNTG; 54 (-) CANNTG; 310 (-) CANNTG; 594 (-) CANNTG; 613 (-) CANNTG; 758 (-) CANNTG; 1105 (-) CANNTG;
	T/G-box	T/GBOXATPIN2	31 (-) AACGTG; 71 (-) AACGTG;
GJ4E51T3 GjbHLH2.5	E-box	EBOXBNNAPA	352 (+) CANNTG; 693 (+) CANNTG; 927 (+) CANNTG; 1102 (+) CANNTG; 1352 (+) CANNTG; 352 (-) CANNTG; 693 (-) CANNTG; 927 (-) CANNTG; 1102 (-) CANNTG; 1352 (-) CANNTG;
		MYCATRD22	1352 (+) CACATG;
GJ2E38T7 GjbHLH3.10	E-box	EBOXBNNAPA	669 (+) CANNTG; 721 (+) CANNTG; 728 (-) CANNTG; 1076 (+) CANNTG; 1110 (+) CANNTG; 1299 (+) CANNTG; 1343 (+) CANNTG; 1477 (+) CANNTG; 669 (-) CANNTG; 721 (-) CANNTG; 728 (+) CANNTG; 1076 (-) CANNTG; 1110 (-) CANNTG; 1299 (-) CANNTG; 1343 (-) CANNTG; 1477 (-) CANNTG;
		MYCATRD22	1299 (+) CACATG;
GJ9P30T15 GjbHLH7.2	E-box	EBOXBNNAPA	70 (+) CANNTG; 503 (+) CANNTG; 583 (+) CANNTG; 764 (+) CANNTG; 1171 (+) CANNTG; 70 (-) CANNTG; 503 (-) CANNTG; 583 (-) CANNTG;

			764 (-) CANNTG; 1171 (-) CANNTG;
		MYCATRD22	503 (+) CACATG;
GJ18P5T14<br>GjbHLH27.3	E-box	EBOXBNNAPA	143 (+) CANNTG; 157 (+) CANNTG; 328 (+) CANNTG;<br>367 (+) CANNTG; 554 (+) CANNTG; 706 (+) CANNTG;<br>1130 (+) CANNTG; 1205 (+) CANNTG; 1373 (+) CANNTG;<br>1383 (+) CANNTG; 1462 (+) CANNTG;<br>143 (-) CANNTG; 157 (-) CANNTG; 328 (-) CANNTG;<br>367 (-) CANNTG; 554 (-) CANNTG; 706 (-) CANNTG;<br>1130 (-) CANNTG; 1205 (-) CANNTG; 1373 (-) CANNTG;<br>1383 (-) CANNTG; 1462 (-) CANNTG;
		MYCATRD22	1373 (-) CACATG ; 1383 (-) CACATG;
